# Deepening the insight into poly(butylene oxide)-*block*-poly(glycidol) synthesis and self-assemblies: micelles, worms and vesicles†

**DOI:** 10.1039/d0ra04274a

**Published:** 2020-06-12

**Authors:** Riccardo Wehr, Jens Gaitzsch, Davy Daubian, Csaba Fodor, Wolfgang Meier

**Affiliations:** University of Basel, Department of Chemistry Mattenstrasse 24a, BPR 1096 4058 Basel Switzerland wolfgang.meier@unibas.ch; Leibniz-Institut für Polymerforschung Dresden e.V. Hohe Strasse 6 01069 Dresden Germany

## Abstract

Aqueous self-assembly of amphiphilic block copolymers is studied extensively for biomedical applications like drug delivery and nanoreactors. The commonly used hydrophilic block poly(ethylene oxide) (PEO), however, suffers from several drawbacks. As a potent alternative, poly(glycidol) (PG) has gained increasing interest, benefiting from its easy synthesis, high biocompatibility and flexibility as well as enhanced functionality compared to PEO. In this study, we present a quick and well-controlled synthesis of poly(butylene oxide)-*block*-poly(glycidol) (PBO-*b*-PG) amphiphilic diblock copolymers together with a straight-forward self-assembly protocol. Depending on the hydrophilic mass fraction of the copolymer, nanoscopic micelles, worms and polymersomes were formed as well as microscopic giant unilamellar vesicles. The particles were analysed regarding their size and shape, using dynamic and static light scattering, TEM and Cryo-TEM imaging as well as confocal laser scanning microscopy. We have discovered a strong dependence of the formed morphology on the self-assembly method and show that only solvent exchange leads to the formation of homogenous phases. Thus, a variety of different structures can be obtained from a highly flexible copolymer, justifying a potential use in biomedical applications.

## Abstract 2

Improved synthesis and well controlled self-assembly of PBO-*b*-PG amphiphilic diblock copolymers led to homogenous phases of micelles, worms and vesicles.

## Graphical abstract

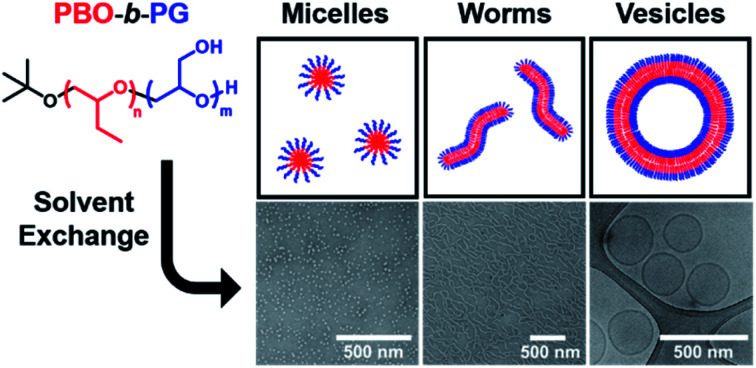

## Introduction

Amphiphilic block copolymers represent a widespread class of biomedically relevant polymers as they exhibit great potential in applications like drug delivery, nanoreactors or cell mimics.^1–3^ They profit from their self-assembly process in aqueous media into numerous different morphologies like spherical micelles, cylindrical micelles (worms), vesicles and related structures.^4–6^ The most prominent hydrophilic block used for those applications is poly(ethylene oxide) (PEO), also referred to as poly(ethylene glycol) (PEG).^7^ Several studies have shown its biocompatibility and stealth effect when conjugated to drugs (PEGylation),^8–11^ making it the gold standard for drug delivery applications. Especially micelles of the triblock copolymer poly(ethylene oxide)-*block*-poly(propylene oxide)-*block*-poly(ethylene oxide) (PEO-*b*-PPO-*b*-PEO), better known as poloxamere or pluronic, have been studied extensively not only for biomedical applications but also as surfactants and emulsion stabilisers in industrial applications.^7,12^

However, PEO suffers from several drawbacks, including the demanding synthesis on a lab scale with its toxic and gaseous monomer ethylene oxide, the lack of functional groups, risk of peroxidation, a possible immune response with anti-PEG antibodies as well as a high protein adsorption on PEGylated drugs.^13–17^ Alternative hydrophilic blocks have been suggested, trying to circumvent the disadvantages of PEO. Among others, poly(oxazoline)s, poly(sarcosine) and poly(glycidol) (PG) have gained increasing interest and seem to be equally biocompatible and protein-resistant while highly functionalisible.^17–20^ PG is of special interest since it has an equally high or even better biocompatibility compared to PEO. It exhibits better antifouling properties due to a reduced protein adsorption,^21–24^ predestining it for the use in biomedical applications.^16,25^ PG is structurally similar to PEO, but exhibits an enhanced hydrophilicity because of the additional hydroxymethyl moieties on every repeating unit. Its synthesis starts from the anionic ring-opening polymerisation (AROP) of easy-to-handle protected glycidol derivatives like 1-ethoxy ethyl glycidyl ether (EEGE), *tert* butyl glycidyl ether (*t*BGE) or allyl glycidyl ether (AGE).^16^ The protection prevents unwanted branching from the hydroxy side groups. Acidic cleavage of the protecting group after polymerisation eventually leads to linear PG.^16,26,27^

Several groups already suggested PG as hydrophilic block in amphiphilic block copolymers,^28–30^ including investigations of their self-assembly behaviour in aqueous solution.^31–35^ However, only a few publications showed the formation of well-defined structures beyond micelles or aggregates. For instance in a study from Rangelov *et al.*, compound vesicle-like structures were obtained by direct dissolution of PG-*b*-PPO-*b*-PG triblock copolymers in water.^36^ Du *et al.* formed micelles and vesicles from a series of poly(butylene oxide)-*block*-poly(glycidol) (PBO-*b*-PG) diblock and triblock copolymers *via* film rehydration and analysed their protein antifouling properties.^21^ In contrast, studies about the influence of different self-assembly techniques as well as the formation of highly defined homogenous self-assembly phases have not been published yet.

The hydrophobic blocks used in aforementioned studies, PPO or PBO, share the same polyether backbone as PEO and PG. PBO, compared to PPO, exhibits a higher hydrophobicity^37^ and is easier to synthesise at a lab scale due to the higher boiling point of the monomer.^38^ Especially its low glass transition temperature of −70 °C ^39^ enables applications of PBO where a high membrane fluidity and flexibility is essential, for example for the encapsulation of enzymes or the insertion of membrane proteins.^40–42^ PBO thus stands out from of a number of alternative commonly used hydrophobic blocks like poly(lactic acid) (PLA) or poly(caprolactone) (PCL). Because of the semi-crystallinity of the latter ones, high temperatures are required during the self-assembly process,^43,44^ which inhibit their combination with many biologically active compounds. Recent studies also highlight the increased cytocompatibility of PBO containing self-assemblies compared to nanoparticles made of more hydrophobic blocks, reinforcing its biomedical relevance.^45–48^

The combination of PG as hydrophilic and PBO as hydrophobic block in amphiphilic block copolymers and their self-assembly will be advantageous for possible biomedical applications. In this work, we present an improved, microwave-based synthesis of PBO-*b*-PG diblock copolymers. We further investigate their self-assembly behaviour in aqueous media by solvent exchange, which is governed by the composition and hydrophilic mass ratio of the copolymers. A large variety of different structures in the nano- and micrometre range is presented and extensively characterised by dynamic and static light scattering (DLS/SLS), TEM, Cryo-TEM and confocal laser scanning microscopy (CLSM). A full insight into the structures and their sizes is given and distinct differences between self-assemblies formed by solvent exchange or film rehydration are revealed.

## Materials and methods

### Materials

All glassware used for the polymerisations was dried overnight at 120 °C prior to use. Potassium *tert* butoxide (KO^*t*^Bu, ≥98%), 1,4,7,10,13,16-hexaoxacyclooctadecane (18-crown-6, ≥99%), 1,2-butylene oxide (BO, 99%), ethyl vinyl ether (EVE, ≥98%), glycidol (96%), calcium hydride (CaH_2_, 95%), potassium (K, chunks in mineral oil, 98%) and naphthalene (Naph, 99%) were purchased from Sigma-Aldrich (Switzerland) and used as received. Dry THF was obtained from an inert solvent purification system PureSolv MD 5 (Inert Technology, USA), dry 1,4-dioxane was purchased from Acros Organics (Belgium). The chemicals were stored under argon in a glovebox (MBraun Labstar, Germany). All other solvents used were in HPLC grade and purchased from JT Baker (USA), VWR (Switzerland) or Scharlau (Germany). Deionised water was obtained from a Milli-Q Q-POD device (Merck, Germany). Dulbecco's PBS buffer was purchased from Bioconcept Ltd (Switzerland).

Potassium naphthalenide (KNaph) was prepared by adding potassium (1.19 g, 30.5 mmol, 1 eq.) to a stirred solution of naphthalene (4.11 g, 32.0 mmol, 1.05 eq.) in dry THF (61 mL, 0.5 mol L^−1^) under argon. EEGE was synthesised following the standard protocol^49^ from glycidol and EVE and dried over CaH_2_. The synthesis protocol and the ^1^H-NMR spectrum (Fig. S1) can be found in the ESI.†

### Methods

#### Nuclear magnetic resonance spectroscopy (NMR)

^1^H NMR spectra were recorded at 295 K in methanol-d_4_ (MeOD) or TMS-free chloroform-d_1_ (CDCl_3_) (Cambridge Isotope Laboratories, USA) on a 500 MHz Advance III NMR spectrometer (Bruker, USA). The device was equipped with a BBFO SP FB standard probe and a default number of 16 scans was used. The water signal in MeOD (4.87 ppm) or the residual solvent peak in CDCl_3_ (7.26 ppm) were used for calibration. Processing of the spectra was performed in MestReNova software (version 11.0, Mestrelab, Spain).

#### Size exclusion chromatography (SEC)

SEC measurements were performed in HPLC grade DMF (Scharlau, Germany) at 60 °C with a flow rate of 1 mL min^−1^. Narrowly distributed poly(methyl methacrylate)s were used as calibration standards. The Viscotek TDA 305 device (UK) was equipped with three SDV Linear S columns (5 μm, 8 × 300 mm, PSS, Germany), a PSS precolumn (SDV, 5 μm, 8 × 50 mm) and a refractive index (RI) detector. The control of the instrument and the analyses of the traces were done in WinGPC UniChrom software (version 8.20, PSS).

#### Differential scanning calorimetry (DSC)

DSC traces were recorded on a DSC 214 Polyma (Netzsch, Austria) under nitrogen atmosphere from −150 to 120 °C with a heating and cooling rate of 10 K min^−1^ from 10 mg of sample in aluminium crucibles. The thermographs were evaluated using Netzsch Proteus software (version 7.1). The second heating curves are shown and were used to analyse the thermal transitions. The glass transition temperatures (*T*_g_) were measured at the inflection points of every curve.

#### Transmission electron microscopy (TEM)

TEM images were recorded on a CM100 transmission electron microscope (Philips, Netherlands) at an acceleration voltage of 80 kV. Formvar-coated 200 mesh copper grids were glow discharged for 30 seconds prior to use. 15 μL of diluted (0.5 mg mL^−1^) self-assembly dispersion were left adsorbing on the grid for one minute and afterwards blotted off with a filter paper. Then, the grid was washed two times with 50 μL sized water drops. Afterwards, a 5 μL sized drop of 2% aqueous uranyl acetate solution was placed on the grid and blotted off immediately. A second 5 μL sized drop of uranyl acetate solution was left adsorbing for 10 seconds and eventually blotted off before imaging the sample. The size of micelles as well as the length and thickness of worms were measured in ImageJ (NIH, USA).

#### Cryogenic transmission electron microscopy (Cryo-TEM)

A Talos electron microscope (Thermo Fisher, USA) equipped with a Gatan 626 Cryo-holder and CETA camera was used for Cryo-TEM imaging. 4 μL of a 4 mg mL^−1^ self-assembly dispersion was adsorbed onto a holey carbon-coated grid (Lacey, Tedpella, USA) and blotted off with Whatman 1 filter paper. The sample was vitrified into liquid ethane at −178 °C using a Leica GP plunger (Leica, Austria). Recording of the micrographs was done at an acceleration voltage of 200 kV and a nominal magnification of 57 000×. A low-dose system (20 e^−^ Å^−2^) was used by maintaining the sample at low temperature. Diameters and membrane thicknesses of vesicles were measured using ImageJ (NIH, USA).

#### Dynamic and static light scattering (DLS/SLS)

Multi-angle light scattering data were recorded on a spectrometer (LS Instruments, Switzerland) which was equipped with a 633 nm He–Ne laser with 21 mW. All experiments were measured at scattering angles between 30 and 135° at 25 °C in round-bottom cuvettes (10 × 0.9–1.0 mm, Boro 3.3). For both DLS and SLS measurements, diluted self-assembly dispersions of 0.05 mg mL^−1^ were used without filtration. Hydrodynamic radii (*R*_h_) were calculated by DLS as a mean value of three independant measurements over the whole angle range using second order cumulant analyses. Polydispersity indices (PDI) were calculated from the 90° DLS measurements. For SLS analyses, the mean intensity of three repetitive measurements was plotted against the respective angle and fitted with a Mie scattering model (MiePlot, UK) for *η* = 1.35 and 5% polydispersity. The radius *R* was obtained from the best fit and transformed into the radius of gyration (*R*_g_) using the formula for spherical structures *R*_g_^2^ = (3/5)*R*^2^.

#### Confocal laser scanning microscopy (CLSM)

CLSM measurements were conducted on a Zeiss LSM 880 confocal laser scanning microscope (Carl Zeiss, Germany) with inverted microscope Zeiss Axio Observer, run by Zen Black software. The device was equipped with a water immersion objective (C-Apochromate 40×/1.2 W Korr FCS M27). Stained giant unilamellar vesicles (GUVs) were excited with a He–Ne laser at 633 nm using a MBS 488/561/633 filter. Each sample was scanned unidirectionally at 512 × 512 pixels with a bit depth of 8 bit and a pinhole aperture of 39 μm at 1 Airy unit. CLSM images were afterwards edited with ImageJ (NIH, USA).

#### Microwave-assisted synthesis

All polymers were synthesised on a Biotage Initiator System (Biotage, Sweden) equipped with Robot Eight. The temperature was monitored with an infrared sensor. The polymerisations were performed at a low absorption level after prestirring for 10 seconds at room temperature.

#### Synthesis of poly(butylene oxide) (PBO)

Potassium *tert* butoxide solution (KO^*t*^Bu, 0.25 mol L^−1^ in 1,4-dioxane, 6.44 mL, 1.61 mmol, 1 eq.) was transferred into a 20 mL microwave vial with a magnetic stirrer in a glovebox. Then, 1,4-dioxane (0.66 mL) and a solution of 18-crown-6 (0.50 mol L^−1^ in dioxane, 1.61 mL, 0.804 mmol, 0.5 eq.) was added. The vial was closed and unloaded together with a syringe filled with 1,2-butylene oxide (BO, 7.00 mL, 80.4 mmol, 50 eq.). BO was added to the reaction mixture through the septum of the lid and the microwave-assisted reaction was immediately started. The first heating step for two minutes at 50 °C was followed by the second step of two minutes at 60 °C and finally 30 min at 70 °C. After cooling to room temperature, the polymerisation was quenched by adding methanol (2 mL). After stirring overnight, the solvents and unreacted monomer were evaporated using a rotary evaporator. The crude polymer was dissolved in *n*-hexane (100 mL) and washed with methanol (100 mL) in order to remove all polar side products. The bottom methanol enriched phase was extracted three times with *n*-hexane (each 100 mL), the four hexane phases were combined and the solvent evaporated on a rotary evaporator. After drying overnight in high vacuum (0.05 mbar) the polymer was stored in a glovebox under argon. 4.69 g (*M*_n_(NMR) = 3100 g mol^−1^, 1.51 mmol, *Đ*(SEC) = 1.05) of colourless, viscous poly(butylene oxide)_42_ (PBO_42_) were obtained.

PBO_42_: ^1^H-NMR (500 MHz, MeOD, 295 K, *δ*, ppm): 0.97 (m, 126H, –CH_2_–C*H*_3_), 1.22 (s, 9H, (*H*_3_C)_3_C–O–), 1.52–1.61 (m, 84H, –C*H*_2_–CH_3_), 3.37 (m, 42H, –CH_2_–C*H*(CH_2_–CH_3_)–O–), 3.4–3.7 (m, 84H, –C*H*_2_–CH(CH_2_–CH_3_)–O–).

#### Synthesis of poly(butylene oxide)-*block*-poly(glycidol) (PBO-*b*-PG)

A typical procedure is shown for PBO_42_-*b*-PG_21_: PBO_42_ (0.5 g, 0.161 mmol, 1 eq.) was transferred into a 5 mL microwave vial with a magnetic stirrer. 1,4-Dioxane (2.18 mL) was added and the vial was closed. A solution of potassium naphthalenide (KNaph, 0.5 mol L^−1^ in THF, 0.322 mL, 0.161 mmol, 1 eq.) was added to the solution dropwise under shaking through the septum of the lid. The addition of KNaph was stopped when the equivalent point was reached, visually indicated by the dark green colour of the solution, which remained for at least two minutes. After stirring for another five minutes, the reaction vial was unloaded together with a syringe filled with EEGE monomer (0.66 mL, 4.03 mmol, 25 eq.). After addition of EEGE through the lid, the microwave-assisted polymerisation was immediately started and run at 70 °C for 2.5 h. After cooling to room temperature, methanol (0.5 mL) was added to quench the reaction. After a minimum of three hours under stirring, the solvents were evaporated using a rotary evaporator. Cleavage of the protecting groups of the crude PBO-*b*-PEEGE copolymer was done in 0.1 M HCl in ethanol (10 mL) for three hours. Afterwards, the acidic solution was neutralised using 1 M NaOH in ethanol. The solvent was partly removed on a rotary evaporator and the same volume Milli-Q water was added. The copolymer solution was then dialysed for two days against a 1 : 1 water : ethanol mixture, using a regenerated cellulose membrane with a MWCO of 1 kDa (RC6, Spectra Por, USA). After five exchanges of the solvent, the solution was dialysed against pure water for two more cycles. Eventually, the copolymer dispersion was lyophilised for two days. 619 mg (*M*_n_(NMR) = 4700 g mol^−1^, 0.132 mmol, *Đ*(SEC) = 1.09) of colourless, solid PBO_42_-*b*-PG_21_ were obtained.

PBO_42_-*b*-PG_35_: ^1^H-NMR (500 MHz, MeOD, 295 K, *δ*, ppm): 0.97 (m, 128H, –CH_2_–C*H*_3_), 1.21 (s, 9H, (*H*_3_C)_3_C–O–), 1.43–1.70 (m, 84H, –C*H*_2_–CH_3_), 3.37 (m, 43H, –CH_2_–C*H*(CH_2_–CH_3_)–O–), 3.45–3.84 (m, 187H, –C*H*_2_–CH(CH_2_–CH_3_)–O–, –C*H*_2_–C*H*(CH_2_–OH)–O–).

#### Self-assembly

Self-assembly into nanoscopic structures *via* solvent exchange was done at polymer concentrations of 4 mg mL^−1^. 8 mg of the copolymer was dissolved in 0.4 mL THF. Under stirring at 300 rpm, 1.6 mL of Milli-Q water or PBS buffer were added using a syringe pump (AL-1000, WPI, USA) with an addition rate of 10 μL min^−1^. After 48 h of stirring, THF was removed *via* dialysis against Milli-Q water or PBS buffer (regenerated cellulose membrane, MWCO 1 kDa, RC6, Spectra Por, USA). The solvent was exchanged four times within two days. Extrusion of the vesicle-containing self-assemblies was done with a Mini Extruder (Avanti Polar Lipids, USA) equipped with a 200 nm polycarbonate membrane in 15 passages.

Self-assembly into nanoscopic structures *via* film rehydration was done by dissolving 8 mg of the copolymer in a 5 mL round-bottom flask. 0.5 mL ethanol were added and the solvent was evaporated on a rotary evaporator at 40 °C, 40 rpm and 140 mbar until a dry polymer film was obtained. The film was rehydrated by adding 2 mL water and stirring for two days at room temperature at 600 rpm.

Self-assembly into macroscopic GUVs was done *via* film rehydration. 50 μL of a 4 mg mL^−1^ polymer solution in ethanol were transferred into a plasma-activated glass vial. The film was deposited within 1 h by evaporating the solvent in a vacuum oven. Aqueous sucrose solution (700 μL, 300 mM) was added into the vial and the film was rehydrated by pipetting five times slowly up and down using an Eppendorf pipette. The dispersion was left standing overnight. Then, 200 μL of the polymer dispersion were transferred into a plasma-activated 8-well chamber plate and diluted with 200 μL PBS buffer. Bodipy dye solution (4 μL, 100 μM) was added, the dispersion was mixed slowly by pipetting up and down and afterwards imaged by CLSM.

## Results and discussion

### Synthesis of the copolymers

**Fig. 1:**
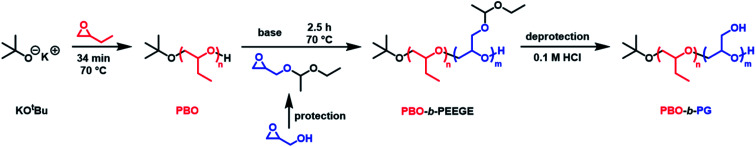
Reaction equation for the synthesis of the amphiphilic diblock copolymer poly(butylene oxide)-*block*-poly(glycidol) (PBO-*b*-PG).

The amphiphilic PBO-*b*-PG diblock copolymers were synthesised in two sequential microwave-assisted AROP. The synthesis protocol involved three steps. The hydrophobic block PBO was synthesised first, followed by the chain extension with the protected glycidol derivative EEGE. Eventually, the acetal protecting group of the PEEGE block was removed in order to obtain the final block copolymer (Fig. 1).

**Fig. 2:**
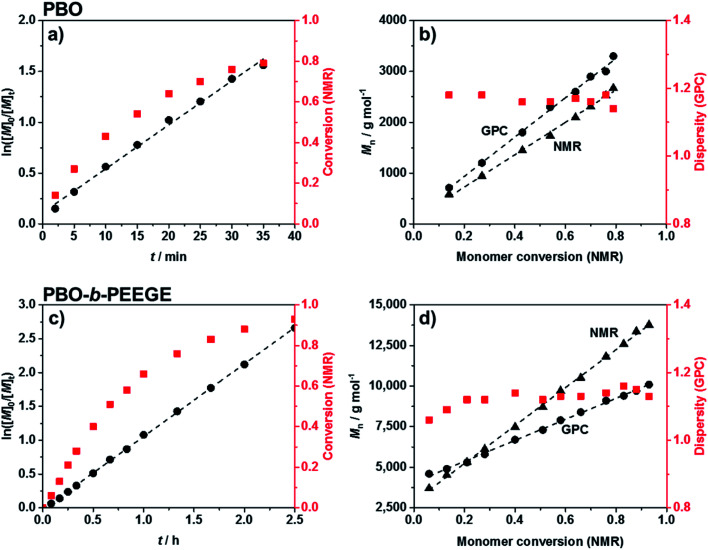
Kinetic analyses for the synthesis of PBO (a and b) and PBO-*b*-PEEGE (c and d). (a) and (c) show the living character of both polymerisations up to high conversions. (b) and (d) show the absence of monomer-consuming side reactions and low dispersities during the syntheses.

Commercially available KO^*t*^Bu was chosen as initiator for the first block. The polymerisation of BO monomer was conducted in a microwave-based reaction at 70 °C. The use of 0.5 equivalents crown ether to solubilise the potassium ions both accelerated the reaction and led to a significantly reduced dispersity.^50,51^ The kinetics of the reaction was analysed *via*^1^H NMR spectroscopy and SEC. The linear increase of ln([*M*]_0_/[*M*]_*t*_) with reaction time *t* (Fig. 2a) represented ideal first-order kinetics. Also the linear increase of the molecular weight with conversion, independently confirmed by NMR and SEC measurements (Fig. 2b), indicated that no unwanted monomer-consuming side reactions took place. The data confirmed a living and well-controlled polymerisation up to a high conversion of 86%. The dispersity remained low during polymerisation and could be decreased even further to 1.05 after purification. The SEC traces of every time point can be found in Fig. S2.† The use of the largest possible microwave vial with a capacity of 20 mL required a stepwise temperature increase from 50 °C over 60 °C, until the actual reaction temperature of 70 °C was reached. A faster heating program would have forced a system shutdown due to the accelerated heating rate. The reaction was quenched by adding methanol in order to obtain hydroxy end groups for the following chain-extension. Compared to time-consuming conventional syntheses at low temperatures (6 days at −15 °C ^52^ or 24 h at 25 °C ^21^) the microwave-based protocol benefited from a drastically reduced reaction time of only 34 min. The quick heating based on permeating microwave irradiation^53^ compared to conventional oilbath-based heat-flow syntheses appeared advantageous for retaining both the high control and living character. Side reactions or broadening of the molecular weight distribution due to the elevated temperatures could not be observed.

Chain-extension of the PBO macroinitiator was done in a similar, microwave-based synthesis using EEGE as an acetal protected glycidol derivative. KNaph was used to activate the PBO homopolymer. A complete deprotonation and thus the titration end point with KNaph was visually recognised as the solution remained clear during deprotonation and the dark green colour of KNaph remained as soon as the reaction was complete.^54^ The use of KNaph as base also benefited from reduced side reactions like chain-transfer, enabling lower dispersities especially at higher molecular weights than comparable metal alkoxides.^55,56^ Kinetic measurements confirmed that the EEGE polymerisation at 70 °C again yielded a perfectly linear growth (Fig. 2c) and retained a living character up to high conversions. Also here both NMR and SEC measurements confirmed the high control. The almost ideal linear increase of molecular weight with EEGE conversion (Fig. 2d) again proved the absence of any monomer-consuming side reactions or the production of high quantities of unwanted homopolymer. Polymerisations were conducted until an EEGE conversion of typically 93% was reached. The high conversion rate was shown not to have a negative effect on the dispersity, which remained low at all time spots (*Đ =* 1.1–1.2, Fig. 2d and S2†) and was even lower after purification. The use of crown ether here was not necessary as the low dispersity was achieved even without on a much shorter time scale of 2.5 hours than conventional PEEGE syntheses (3 or 4 days at 50 °C ^28,29^ or 24 h at 25 °C ^21^). Cleavage of the acetal protecting group on every PEEGE repeating unit was done without previous purification of the copolymer in 0.1 M HCl. The low concentration of acid was necessary in order to prevent the *tert* butoxy end group from being cleaved as well, a risk more pronounced for higher acid concentrations.^57^ A possible loss of end groups would have been detectable *via* NMR spectroscopy by an apparent increased degree of polymerisations (DP) of both blocks. As this was not observed, it was concluded that the end groups stayed intact. After neutralisation, the mixtures were purified by dialysis to give the final diblock copolymers PBO_42_-*b*-PG_77_ (*Đ* = 1.10), PBO_42_-*b*-PG_35_ (*Đ* = 1.09) and PBO_42_-*b*-PG_21_ (*Đ* = 1.09) (Table 1).

**Table 1:** Composition, molecular weight, hydrophilic mass ratio *f* (*f* = *M*_n_(PG)/*M*_n_(PBO-*b*-PG)), dispersity and radii of the self-assembled structures of the PBO-*b*-PG diblock copolymers presented in this work. The top three entries correspond to copolymers, which form pure phases of self-assemblies and which are discussed in detail. The bottom four entries correspond to mixed phases of self-assemblies for comparison. Their characterisation is shown in Fig. S4–S7^a^

Composition^a^	*M*_n_^a^/g mol^−1^	*f*-ratio^a^	*Đ* ^b^	Structure	*R*_h_^c^/nm	PDI^c^	*R*_g_^d^/nm	*ρ* = *R*_g_/*R*_h_
PBO_42_-*b*-PG_77_	8800	0.65	1.10	Micelles	11.2 ± 0.6	0.40 ± 0.05	—	—
PBO_42_-*b*-PG_35_	5700	0.46	1.09	Worms	26.0 ± 2.0	0.33 ± 0.02	—	—
PBO_42_-*b*-PG_21_	4700	0.33	1.09	Vesicles	108 ± 11^e^	0.15 ± 0.05^e^	101^e^	0.93^e^
PBO_36_-*b*-PG_59_	7000	0.62	1.11	Mixed	20.1 ± 2.0	0.47 ± 0.01	—	—
PBO_30_-*b*-PG_38_	5100	0.56	1.09	Mixed	12.1 ± 0.4	0.45 ± 0.01	—	—
PBO_50_-*b*-PG_18_	5000	0.27	1.04	Mixed	194 ± 3	0.37 ± 0.02	—	—
PBO_67_-*b*-PG_14_	5900	0.17	1.08	Mixed	120 ± 6	0.31 ± 0.01	—	—

a Values determined by (a) ^1^H-NMR, (b) SEC, (c) DLS and (d) SLS, for vesicles after extrusion through a 200 nm filter (e).

### Copolymer characterisation

**Fig. 3:**
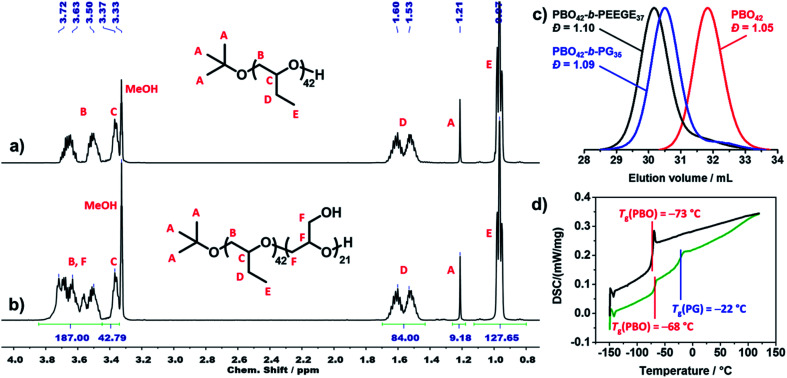
^1^H NMR spectrum of (a) PBO_42_ and (b) PBO_42_-*b*-PG_21_ in MeOD (500 MHz, 295 K, 16 scans). (c) SEC traces of PBO_42_ homopolymer (red), PBO_42_-*b*-PEEGE_37_ before cleavage of the protecting group (black) and PBO_42_-*b*-PG_35_ after cleavage of the protecting group (blue) in DMF, showing the low dispersities in all steps (RI detector signal). (d) DSC traces (heating rate 10 K min^−1^) of PBO_42_ homopolymer (black) and PBO_42_-*b*-PG_35_ diblock copolymer (green), showing distinct glass transition temperatures for both blocks of the copolymer.

All polymers were characterised using ^1^H NMR spectroscopy, SEC and DSC. ^1^H NMR spectra were used to determine the average composition and molecular weight of all polymers. Fig. 3a shows the ^1^H NMR spectrum of PBO_42_. The signal of the *tert* butoxy end group (protons A) was used as integration reference to determine the degree of polymerisation (DP) of the PBO macroinitiator. As this signal was overlaid in the spectrum of the crude PBO-*b*-PEEGE (Fig. S3†), the methylene signal of the PBO side groups (protons D) was used as integration reference and set to a DP of 42 for the copolymers before deprotection. In order to be consistent, this signal was retained as reference also for the spectra of the cleaved and purified copolymers. A representative spectrum of PBO_42_-*b*-PG_21_ is shown in Fig. 3b. The DP of the PG block was determined with 21 units based on the signals between 3.4 and 3.8 ppm. The average molecular weights of all copolymers as well as the hydrophilic mass ratios *f* (*f* = *M*_n_(PG)/*M*_n_(PBO-*b*-PG)) are summarised in Table 1.

Fig. 3c shows representative SEC traces of PBO_42_ homopolymer (red), PBO_42_-*b*-PEEGE_37_ before cleavage of the protecting groups (black) and the final PBO_42_-*b*-PG_35_ (blue). After addition of the PEEGE block, the trace of the PBO_42_ homopolymer (*Đ* = 1.05) was shifted to higher molecular weights while the dispersity remained low (*Đ* = 1.10), showing a successful chain-extension. Cleavage of the protecting groups resulted in a shift to smaller molecular weights with the dispersity remaining at its low level of 1.09. The small low molecular weight shoulders in the traces of PBO_42_-*b*-PEEGE_37_ and PBO_42_-*b*-PG_35_ corresponded to small amounts of PEEGE and PG homopolymer, which was probably caused by a slight overtitration with KNaph or by transfer reactions. Especially for copolymers with shorter hydrophilic blocks, it was possible to remove remaining PG homopolymer by dialysis to a large extend (Fig. S7†), explaining the slightly decreased DP of PG compared to the DP of PEEGE. Remaining PBO homopolymer could not be observed, indicating a complete deprotonation and chain-extension of all PBO chains.

Thermal transitions of the copolymers were analysed by DSC in order to elucidate their suitability for applications which require flexible and amorphous blocks. The representative traces of PBO_42_ and PBO_42_-*b*-PG_35_ are shown in Fig. 3d. The PBO block of the copolymer exhibited a *T*_g_ of −68 °C, which was in agreement to the measured *T*_g_ of the PBO homopolymer, −73 °C, as well as the literature value of −70°.^39^ The *T*_g_ of the PG block was detectable at −22 °C, which matched the literature-known values of PG homopolymer between −32 °C ^58^ and −15 °C ^59^ depending on end groups and chain lengths. Also the DSC traces of the other two copolymers (Fig. S8†) exhibited a similar behaviour with *T*_g_'s in the expected ranges. The *T*_g_'s of the PG blocks increased with increasing DP from −23 °C for DP 21 over −22 °C for DP 35 to −14 °C for DP 77.^60^ The appearance of two separate glass transition areas below room temperature as well as the absence of further thermal transitions like crystallinisation or melting peaks confirmed the presence of two distinct, immiscible and amorphous blocks.

### Self-assembly

**Fig. 4:**
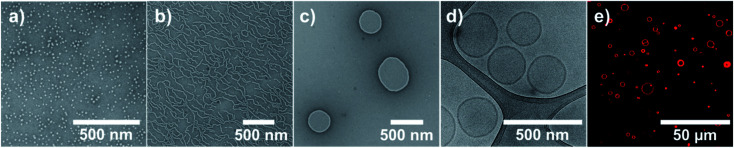
TEM, Cryo-TEM and CLSM images of the nano- and macroscopic self-assemblies: (a) TEM image of micelles formed by solvent exchange from PBO_42_-*b*-PG_77_, (b) TEM image of worms formed by solvent exchange from PBO_42_-*b*-PG_35_, (c) TEM and (d) Cryo-TEM images of polymersomes formed by solvent exchange after extrusion from PBO_42_-*b*-PG_21_, (e) CLSM image of GUVs formed by film rehydration from PBO_42_-*b*-PG_21_.

Self-assembly was conducted *via* solvent exchange in order to obtain pure phases of nanoscopic micelles, worms and vesicles. Solvent exchange was done by adding water to a solution of copolymer in THF and removal of the organic solvent *via* dialysis (Fig. S9†). A slow addition of water (10 μL min^−1^) together with moderate stirring (300 rpm) was essential in order to obtain pure self-assembly phases. Faster addition rates (40 μL min^−1^ or more) led to precipitation or impure phases. The standard protocol involved a stirring time over two nights before the dialysis was started. However, a kinetic analysis (Fig. S10†) indicated that already six hours after the addition of water had started, the assemblies were completely formed. The kinetic studies showed as well that the removal of organic solvent led to no structural changes of the assemblies, neither in their sizes nor in the morphology seen by TEM imaging. The absence of morphological changes over the next days of stirring indicated that the self-assembly process was complete and the structures were stable. All nanoscopic self-assemblies were analysed *via* TEM and DLS, the polymersomes additionally with SLS and Cryo-TEM (Fig. 4 and Table 1).

Self-assembly of PBO_42_-*b*-PG_77_ (hydrophilic mass ratio *f* = 65%) led to the formation of pure phases of spherical micelles (Fig. 4a and S11†). Their radius was determined from several TEM images by measuring the surface area of the flattened micelles. The mean radius was then calculated back to be 6.2 ± 1.3 nm (Fig. S12†). DLS measurements, however, suggested a hydrodynamic radius of 11.2 ± 0.6 nm. The surprisingly high PDI of 0.40 ± 0.05 could be explained by artefacts of diffusing dust particles as the samples could not be filtered before the measurements. The discrepancy between the two radii could be explained by the different measuring techniques. TEM showed flattened self-assemblies due to the applied vacuum, also the automatic calculation of the area was based on contrast between stained micelle and background and thus likely to be inaccurate. In contrast, DLS measurements were based on the diffusion of particles in solution together with their solvating molecules. A higher radius here was thus expected and conclusive with literature reports.^60,61^ In order to compare the measured radii with theoretical considerations, the end-to-end distance *r* in a random coil-like confirmation as well as the maximum chain length *l*_max_ in stretched confirmation of the PBO block were estimated. The calculations are shown in Chapter 8 of the ESI.†*r* was found to be 2.32 nm, *l*_max_ 15.0 nm. The radii determined by DLS and TEM lay between those theoretical values, hence we assumed the PBO chains to assemble in a stacked elongated rather than a random coil-like conformation. Considering those facts, the measured radii were consistent and in the expected order of magnitude.

The copolymer PBO_42_-*b*-PG_35_ with *f* = 46% formed pure wormlike phases (Fig. 4b and S13†) with an *R*_h_ of 26.0 ± 2.0 nm and a PDI of 0.33 ± 0.02. Several worms were analysed from TEM images, revealing typical worm lengths between 100 and 700 nm and an average worm thickness of 15.9 ± 1.3 nm (Fig. S14†). The half of this value, 8.0 ± 0.7 nm, corresponded to a monolayer and was hence very much consistent to the radius determined for the micelles. It laid again between the theoretical lengths of the PBO block in a random coil (*r* = 2.32 nm) and in a stretched conformation (*l*_max_ = 15.0 nm). Also here, an elongated chain conformation was assumed, originating from the crowded environment in the membrane.

Nanometre-sized vesicles were obtained by the self-assembly of PBO_42_-*b*-PG_21_ (*f* = 33%). The polymersomes were formed in homogenous phases, however, showed a large size polydispersity (PDI = 0.34 ± 0.01). Typical diameters were between 50 and 700 nm according to TEM and Cryo-TEM images, which proved the formation of polymersomes (Fig. S15 and S16†). DLS measurements revealed an *R*_h_ of 127 ± 16 nm. In order to confirm the formation of vesicles also *via* light scattering, SLS measurements were conducted. Following the Mie model, a radius of gyration *R*_g_ of 147 nm was calculated (Fig. S17†). Dividing *R*_g_ by *R*_h_ led to the particle scattering factor *ρ*, which is a measure for the morphology of the formed structure.^62^ A value of *ρ* = 1.0 represents an ideal hollow sphere with infinitely thin shell. A full sphere is expected to give *ρ* = 0.775. Hence, a particle scattering factor of *ρ* = 1.0 is regarded as ideal for polymersomes. For the vesicles presented here a form factor of *ρ* = 1.16 was found. This surprisingly high value presumably originated from the high polydispersity, which prevented a more accurate assessment of *R*_g_. In order to narrow down the size distribution and enable a more reliable characterisation, the sample was extruded through a 200 nm membrane. DLS revealed then an *R*_h_ of 108 ± 11 nm with a drastically decreased PDI of 0.15 ± 0.05 and SLS an *R*_g_ of 101 nm (Fig. S17†). The resulting particle scattering factor of *ρ* = 0.93 indicated the presence of hollow polymersomes with a thicker membrane, which was expected for polymersomes of this size. TEM and Cryo-TEM images were used to confirm the presence of vesicles. They showed homogenous phases with smaller polymersomes compared to the images before extrusion (Fig. 4c, d, S18 and S19†). The diameter and membrane thickness were determined by Cryo-TEM (Fig. S20†). Measuring several vesicles led to a mean diameter of 258 ± 82 nm. Despite a more uniform appearance after extrusion, the error showed that the sizes were still not completely uniform, ranging from 100 up to 450 nm. The membrane thickness was determined to be 12.5 ± 1.5 nm, measuring in total 200 spots on several vesicles. Half of this value, 6.3 ± 0.8 nm, again corresponded to a monolayer of the PBO block. The measured value was very much consistent with the theoretical numbers for coil-like and stretched conformations, assuming again a stacked elongated chain conformation. Comparing all three radii, the worms showed the most stretched conformation of all unimers, which could be explained by the crowded environment of a linearly extended self-assembly structure with no curvature in this dimension.

In order to enable a use of those vesicles in physiological environments, we tested the self-assembly of PBO_42_-*b*-PG_21_ not only in water, but also in PBS buffer. Without extrusion, DLS measurements revealed an *R*_h_ of 129 ± 15 nm and a PDI of 0.35 ± 0.01. Both values were similar to the values obtained for the self-assembly in water. SLS confirmed the presence of vesicles with an *R*_g_ of 136 nm and a particle scattering factor *ρ* of 1.05, close to the ideal value of 1.0 for polymersomes (Fig. S21†).^62^ The difference of *R*_g_ and thus *ρ* compared to water (*R*_g_ = 147 nm, *ρ* = 1.16) was probably caused by the high polydispersity of both samples, again disturbing a more accurate determination of the actual values. TEM images also suggested vesicular structures with comparable morphologies and sizes as in water. Only minor aggregates and artefacts inside the flattened polymersomes were visible. The latter ones presumably originated from electrostatic interactions upon drying, caused by salt ions in between the unimers (Fig. S22†). In summary, both water and PBS buffer were considered to be equally suitable as dispersion medium.

As expected from literature,^63^ the morphology of self-assembled structures depended on the solvent used to dissolve the vesicle-forming copolymer PBO_42_-*b*-PG_21_. Several self-assembly tests were run under the same conditions, but using other water-miscible organic solvents than THF, which were able to dissolve both blocks: 1,4-dioxane, acetone, methanol, ethanol and isopropanol (Fig. S23–S28†). Pure vesicular structures were obtained only in the case of THF. All other solvents showed mixed and inhomogenous phases usually consisting of vesicles, micelles, worms or undefined aggregates. It is presumed that the different solvatisation of the copolymer during the assembly as well as the solvent–water interactions determined the packing parameters of the unimers and curvature of the formed membrane.^64^ The preferred vesicular structure governed by the surrounding solvent was apparently only preserved when THF was used, even though the residual solvent was removed afterwards.

In contrast to nanoscopic vesicles prepared *via* solvent exchange, the same copolymer PBO_42_-*b*-PG_21_ (*f* = 33%) was able to form microscopic giant unilamellar vesicles (GUVs) *via* film rehydration. The copolymer film was rehydrated with aqueous sucrose solution. Upon dilution with PBS buffer, the GUVs sank down to the bottom of the vial due to the higher density of the sucrose solution in the inside of the GUVs. The isomolarity of sucrose and PBS buffer prevented rupture of the vesicles due to a possible osmotic shock during dilution.^65^ The membrane of the GUVs was fluorescently labelled using bodipy, a hydrophobic dye which diffused into the hydrophobic core of the polymer membrane, and imaged using CLSM. Mostly GUVs with diameters between 2 and 20 μm were formed, but also multilamellar or multicompartment vesicles or aggregates (Fig. 4e and S29†). These results underlined the broad variety of self-assembly structures that PBO-*b*-PG amphiphilic block copolymers were able to form.

### Discussion of the self-assembly results

**Fig. 5:**
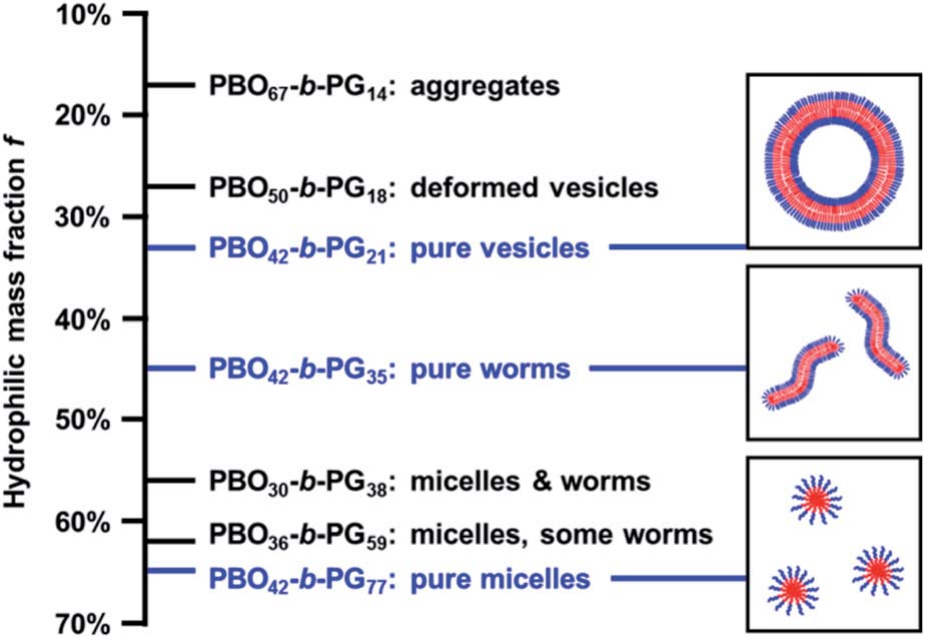
Self-assembly phase diagram of PBO-*b*-PG structures formed by solvent exchange: depending on the composition and hydrophilic mass fraction *f*, pure or mixed phases are formed. The pure phases (blue) are represented by schematic sketches on the right.

The copolymers presented in this work displayed an ideal example of the established self-assembly theory, regarding the correlation between composition, *i.e.* hydrophilic mass fraction *f*, and self-assembled structure.^1,2,6,66^ With decreasing *f*-ratio, the transition from micelles (*f* = 65%) over worms (*f* = 46%) to vesicles (*f* = 33%) was observed. The *f* values observed here exactly matched reported target values representating pure phases of micelles (>45%), worms (<50%) and vesicles (35% ± 10%).^67^ Consequently, copolymers synthesised using adjusted PBO macroinitiators with intermediate *f*-ratios showed mixed phases (Table 1 and Fig. 5). For example, the copolymer PBO_36_-*b*-PG_59_ (*f* = 62%, Fig. S30†) formed mostly micelles with some worms or elongated micelles. PBO_30_-*b*-PG_38_ (*f* = 56%, Fig. S31†) formed a mixed phase with both micelles and worms. Lower hydrophilic ratios than 33% resulted in inhomogenous vesicular phases with dimeric and deformed structures (PBO_50_-*b*-PG_18_, *f* = 27%, Fig. S32†) or undefined aggregates (PBO_67_-*b*-PG_14_, *f* = 17%, Fig. S33†). In particular, the comparison of the copolymers with *f* = 62% and *f* = 65% suggested that even small changes in the average composition already led to significant differences in the formed morphology. However, morphological differences between comparable copolymer batches could also be explained by the possible presence of small impurities, which could have affected the chain packing during self-assembly. Due to the good control and reproducibility of the polymerisations, it was possible to exactly hit the targeted *f* values in order to systematically scan for the compositions which allowed the formation of the highly homogenous self-assembly phases presented above.

**Fig. 6:**
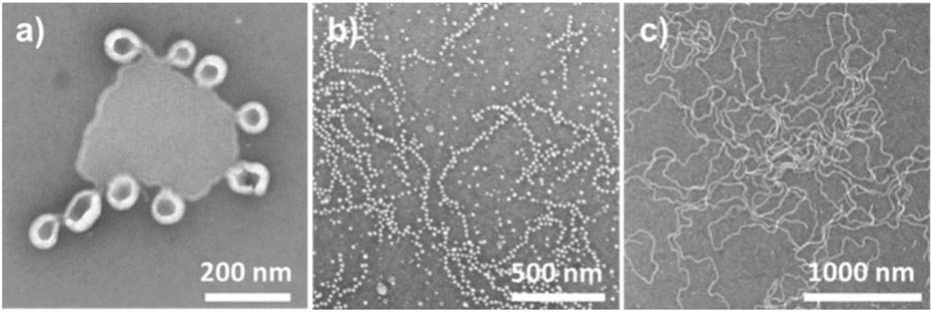
TEM images of the nanoscopic self-assemblies formed by film rehydration from (a) PBO_42_-*b*-PG_21_ (*R*_h_ = 713 ± 103 nm, PDI = 0.54 ± 0.39), (b) PBO_42_-*b*-PG_77_ (*R*_h_ = 63.3 ± 0.5 nm, PDI = 0.37 ± 0.01), (c) PBO_42_-*b*-PG_35_ (*R*_h_ = 68.9 ± 1.0 nm, PDI = 0.43 ± 0.01).

The homogeneity of micelles, worms and vesicles underlined the power of solvent exchange as self-assembly technique. Both light scattering as well as TEM imaging revealed pure phases without aggregates, if copolymer composition, solvent and self-assembly protocol were optimised. Self-assembly by film rehydration of the copolymers presented here led to inhomogenous phases. In case of the vesicle-forming copolymer PBO_42_-*b*-PG_21_, TEM images showed the presence of compound vesicles or polymer films (Fig. 6a and S34†). Those seemed to undergo a transition into vesicular structures, which was, however, not complete. Also micelles formed from PBO_42_-*b*-PG_77_ (*f* = 65%) by film rehydration were not uniformly distributed, but partly aligned to chain-like structures, indicating an incomplete separation (Fig. 6b and S35†). Worms in similar morphology, but distinctly longer, were obtained from PBO_42_-*b*-PG_35_ by film rehydration (Fig. 6c and S36†). Especially in contrast to self-assemblies formed by film rehydration from PBO-*b*-PG diblock and PG-*b*-PBO-*b*-PG triblock copolymers, recently published by Du *et al.*,^21^ the filtration of impurities like aggregates was not necessary in the solvent exchange protocol discussed in here. A comparison of both studies also suggested, that self-assembly *via* solvent exchange could make much more use of the defined composition of the block copolymers as it led to distinct phases. As mentioned above, small differences in the hydrophilic fraction led to rather big differences in the obtained morphologies, including mixed phases. In case of film rehydration, big differences in the composition seemed to have only a minor effect on the structures formed, as Du *et al.* obtained micelles with hydrophilic block ratios between 49 and 79% and vesicles between 18 and 29%.^21^ The latter numbers are rather surprising, as comparable values in the case of solvent exchange only led to impure phases and aggregates. Du *et al.* also observed the formation of mixed cylindrical and vesicular phases for a copolymer with a hydrophilic fraction of 40%.^21^ This was conclusive with our findings as their stated *f*-fraction was set in between the ones presented in here to form vesicles and worms.

It can be suggested that the differences between the two formation methods are caused by the kinetics during the self-assembly process.^4,63^ We hypothesise the following: film rehydration as top-down method did not allow for a sufficient solubilisation of the unimer chains from a bulk material to self-assemble into a well-defined and stable structure. Not every unimer was solely removed from the film, but rather scratched off with surrounding material, leading to kinetically trapped aggregates. In contrast, solvent exchange started from completely dissolved and flexible unimer chains, which were allowed to find a favoured surrounding on a larger time scale due to the slow addition of water. As a bottom-up assembly, it enabled a more distinct morphology which represented the preferred steric surrounding for every unimer chain. This led to pure phases if all conditions were matched, but also to mixed phases for intermediate compositions together with a lower chance of only kinetically frozen aggregates.

## Conclusion

A series of PBO-*b*-PG amphiphilic diblock copolymers was synthesised in microwave-assisted reactions. Kinetic measurements confirmed the high control and reproducibility of the polymerisation at a short time scale of maximum 2.5 h. Self-assembly *via* solvent exchange led to the formation of well-defined structures in pure phases as proven by light scattering and TEM measurements. The formed structures followed the established self-assembly theory by undergoing a transition from micelles over worms to nanoscopic vesicles with decreasing hydrophilic mass fraction. Also microscopic GUVs were prepared, showing the versatility of possible structures. A comparison between solvent exchange and film rehydration results explained the differences between both techniques. It was shown that only by solvent exchange, stable and well-defined structures were formed when the appropriate conditions like solvent, composition of the copolymer and protocol were applied. The results extended the previous work from Du *et al.*^21^ regarding synthesis and self-assembly into homogenous phases of a variety of different structures. The self-assemblies presented here benefit from their purity and reproducibility. Because of the high biocompatibility and flexibility of the copolymer chains, the use in biomedical applications will be advantageous. Nanoscopic vesicles predistine them for a use as nanoreactors or drug delivery systems, whereas the GUVs enable a possible mimicking of cell-like architectures.

## Conflicts of interest

There are no conflicts to declare.

## Supplementary Material

RA-010-D0RA04274A-s001
