# Supplementary material for: Deepening the insight into poly(butylene oxide)-block-poly(glycidol) synthesis and self-assemblies: micelles, worms and vesicles
Source: RSC Adv. 2020 Jun 12;10(38):22701–11. doi: 10.1039/d0ra04274a (PMC9054609; doi:10.1039/d0ra04274a)
Supplement: RA-010-D0RA04274A-s001 [file RA-010-D0RA04274A-s001.pdf]

Electronic Supplementary Material (ESI) for RSC Advances.
This journal is © The Royal Society of Chemistry 2020

Deepening the Insight into Poly(butylene oxide)-*block*-poly(glycidol) Synthesis and Self-assemblies: Micelles, Worms and Vesicles

Riccardo Wehr,[1] Jens Gaitzsch,[1, 2] Davy Daubian,[1] Csaba Fodor,[1] Wolfgang Meier[1, *]

[1] University of Basel, Department of Chemistry, Mattenstrasse 24a, BPR 1096, 4058 Basel, Switzerland

[2] Leibniz-Institut für Polymerforschung Dresden e.V., Hohe Strasse 6, 01069 Dresden, Germany

* Corresponding Author: wolfgang.meier@unibas.ch

Supporting Information

Contents

1. Monomer and Polymer Synthesis and Characterisation 2
2. Self-assembly protocol 6
3. Characterisation of Solvent Switch Self-Assemblies 7
4. Effect of Buffer and Different Solvents 11
5. Self-Assembly into GUVs 14
6. Mixed Phases of Intermediate Copolymer Compositions 14
7. Self-Assembly *via* Film Rehydration 16
8. Chain Length in Coil-like and Stretched Conformation 17

1. Monomer and Polymer Synthesis and Characterisation

Synthesis of 1-ethoxy ethyl vinyl ether (EEGE)

EEGE was synthesised following the common standard protocol.[1] Glycidol (40.0 g, 0.54 mol, 1 eq) was dissolved in ethyl vinyl ether (EVE, 150 g, 2.08 mol, 3.85 eq), followed by the addition of *para*-toluene sulfonic acid monohydrate (1.03 g, 5.40 mmol, 0,01 eq) over 15 min under ice cooling. The solution was stirred overnight while allowing to heat up to room temperature. The crude product mixture was washed three times with saturated aqueous sodium hydrogencarbonate solution. The organic phase was dried with magnesium sulfate and remaining EVE was evaporated on a rotary evaporator. The product was distilled in vacuum and the fraction evaporating at 65–72 °C (0.27 mbar) was collected (57.4 g, 0.39 mol, yield: 73%) and dried over calcium hydride before storing it under argon.

EEGE: ^{1}H-NMR (500 MHz, $CDCl_3$, 295 K, δ, ppm): 1.18 (t, 3H, $-CH_2-CH_3$), 1.30 (m, 3H, $-CH-CH_3$), 2.56–2.65 (m, 1H, epoxy-$CH_{2,\,cis}$), 2.78 (m, epoxy-$CH_{2,\,trans}$), 3.13 (m, 1H, epoxy-CH), 3.38–3.81 (m, 4H, $-CH_2-O-CH(CH_3)-O-CH_2-CH_3$), 4.74 (m, 1H, $O-CH(CH_3)-O-$).

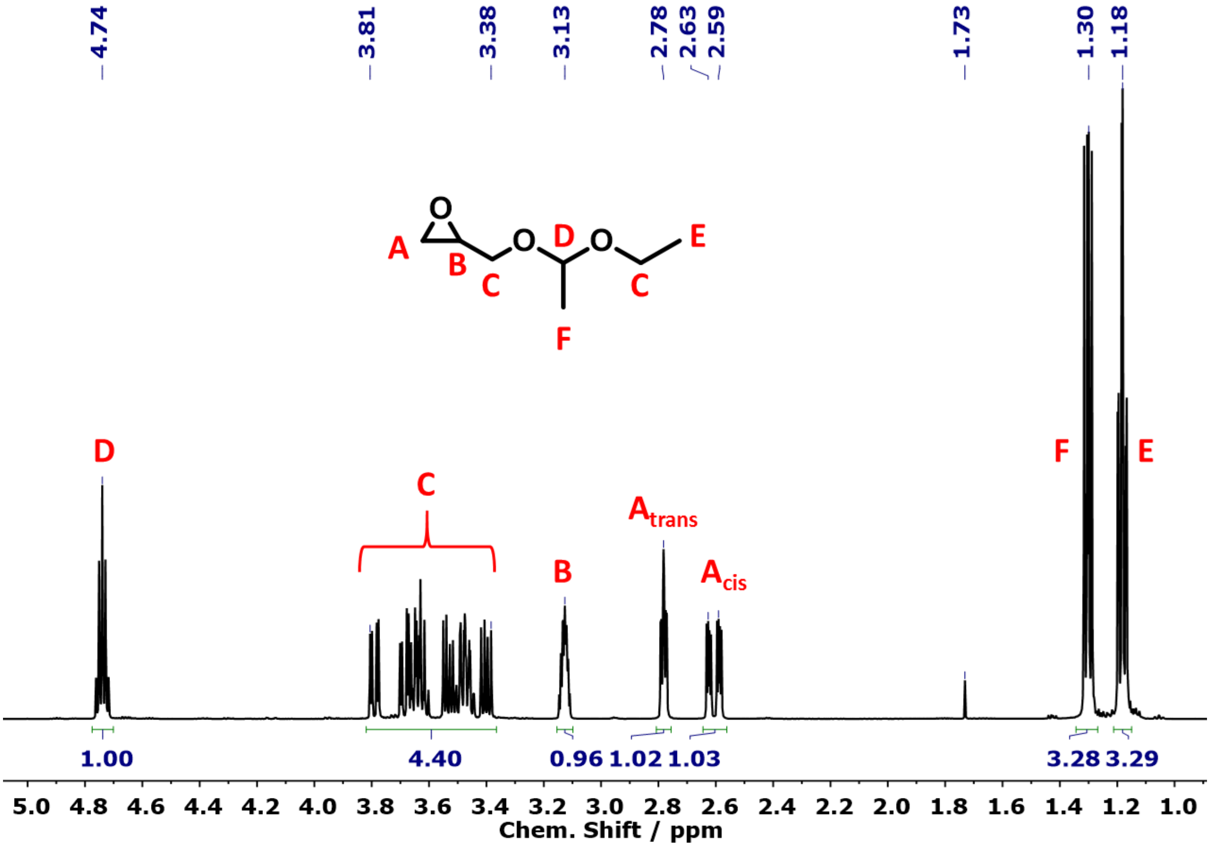

Figure S1: ^{1}H-NMR spectrum of EEGE monomer in $CDCl_3$.

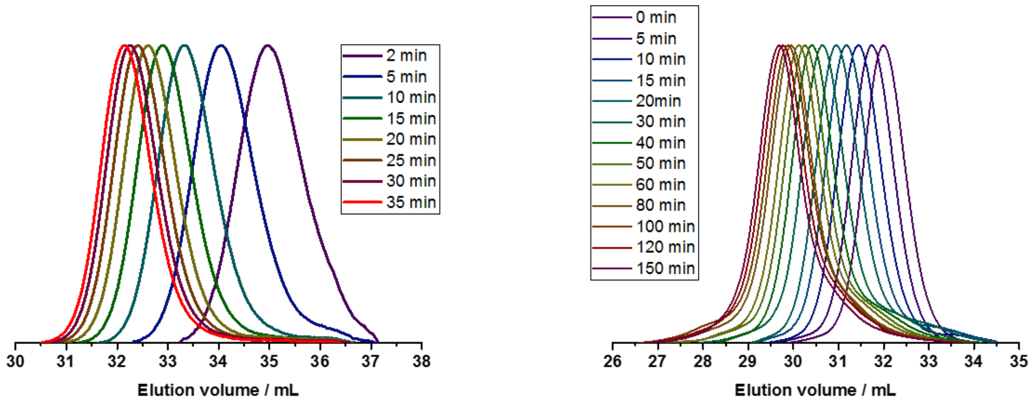

Figure S2: SEC traces of the kinetic measurements of PBO (left) and PBO-*b*-PEEGE (right) in DMF. The latter one shows the development of a small PEEGE homopolymer shoulder with increasing time.

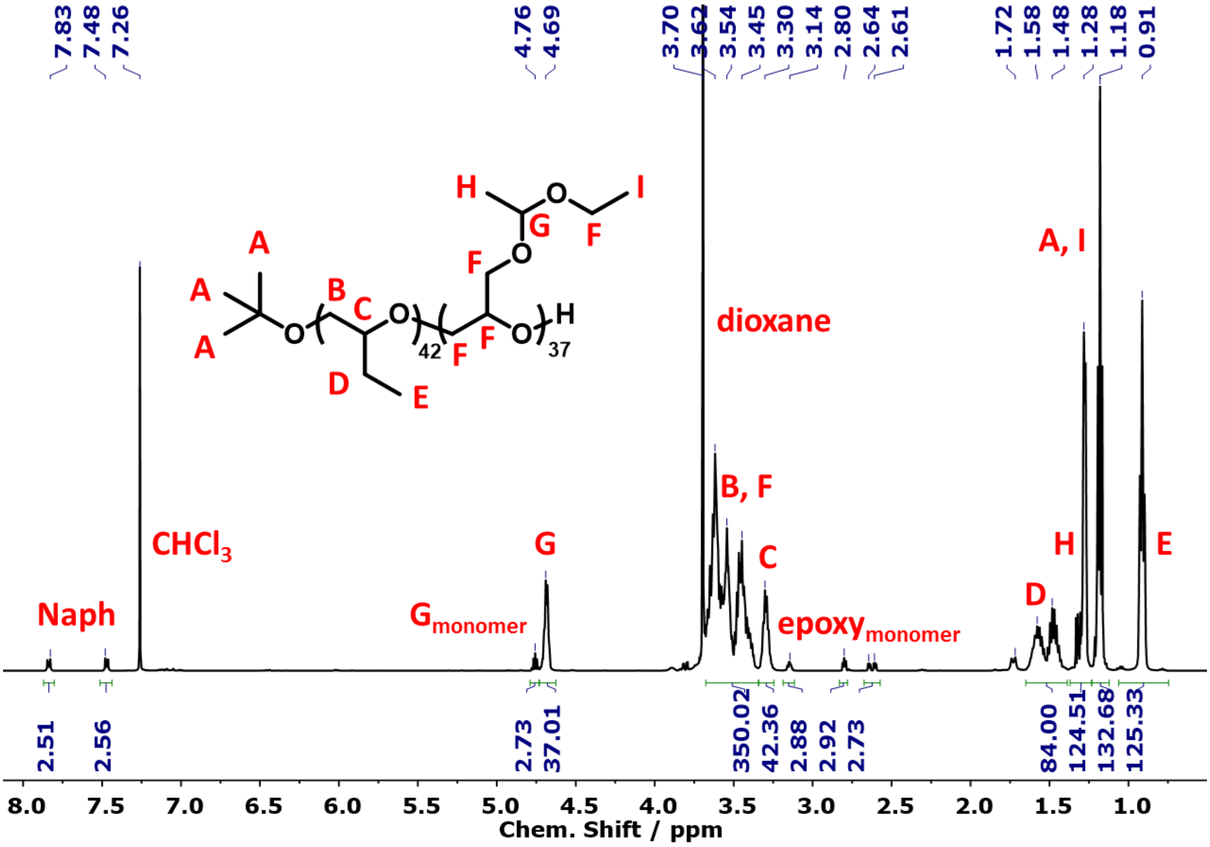

Figure S3: ^{1}H-NMR spectrum of crude PBO_{42}-*b*-$PEEGE_{37}$ in $CDCl_3$, as the copolymer was not purified before the cleavage of the protecting groups. Using the *tert*. butoxy group as integration reference was not possible due to the overlaid signal, so the PBO peak D was set to its integral according to the previously determined degree of polymerisation of 42. The conversion was calculated by the integrals of the signals G of monomer and polymer: conversion = 1-($G_{monomer}$/(G+$G_{monomer}$) = 93%.

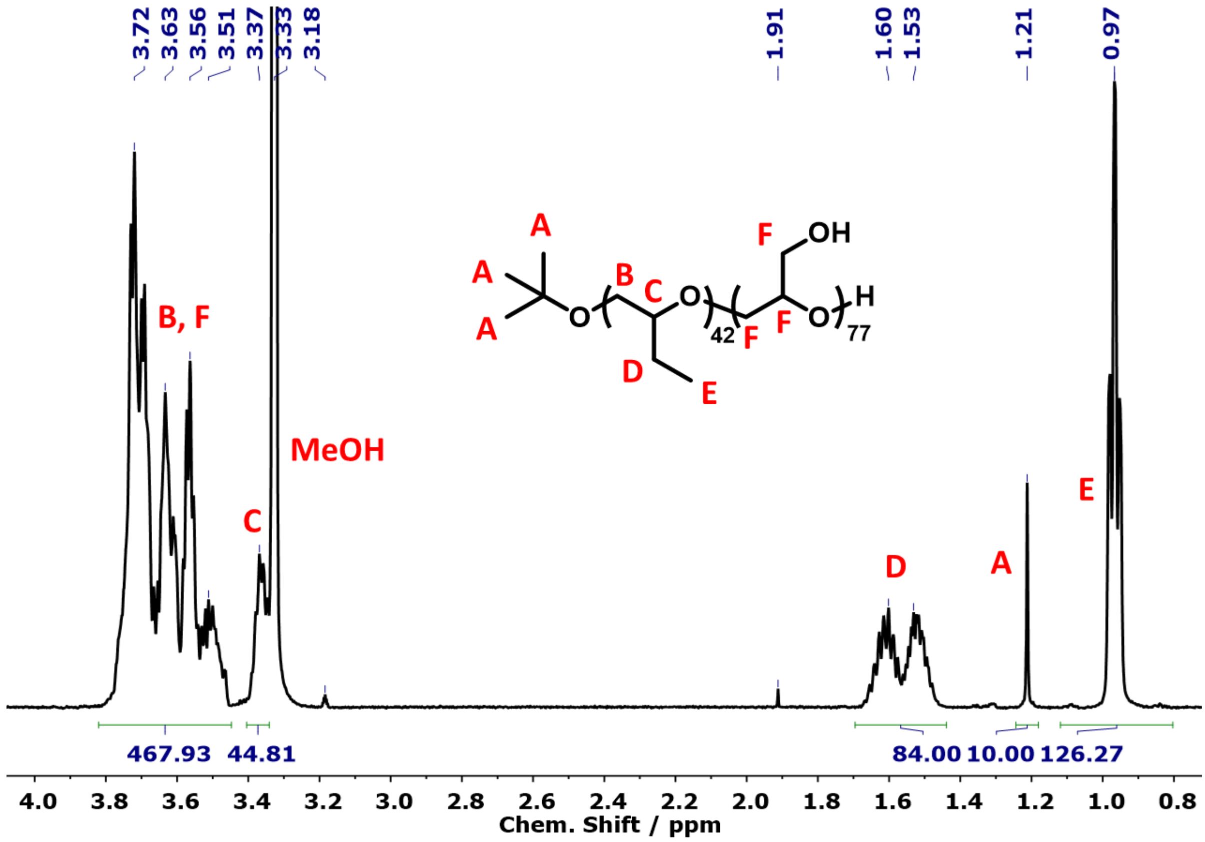

Figure S4: ^{1}H-NMR spectrum of PBO_{42}-*b*-PG_{77} in MeOD.

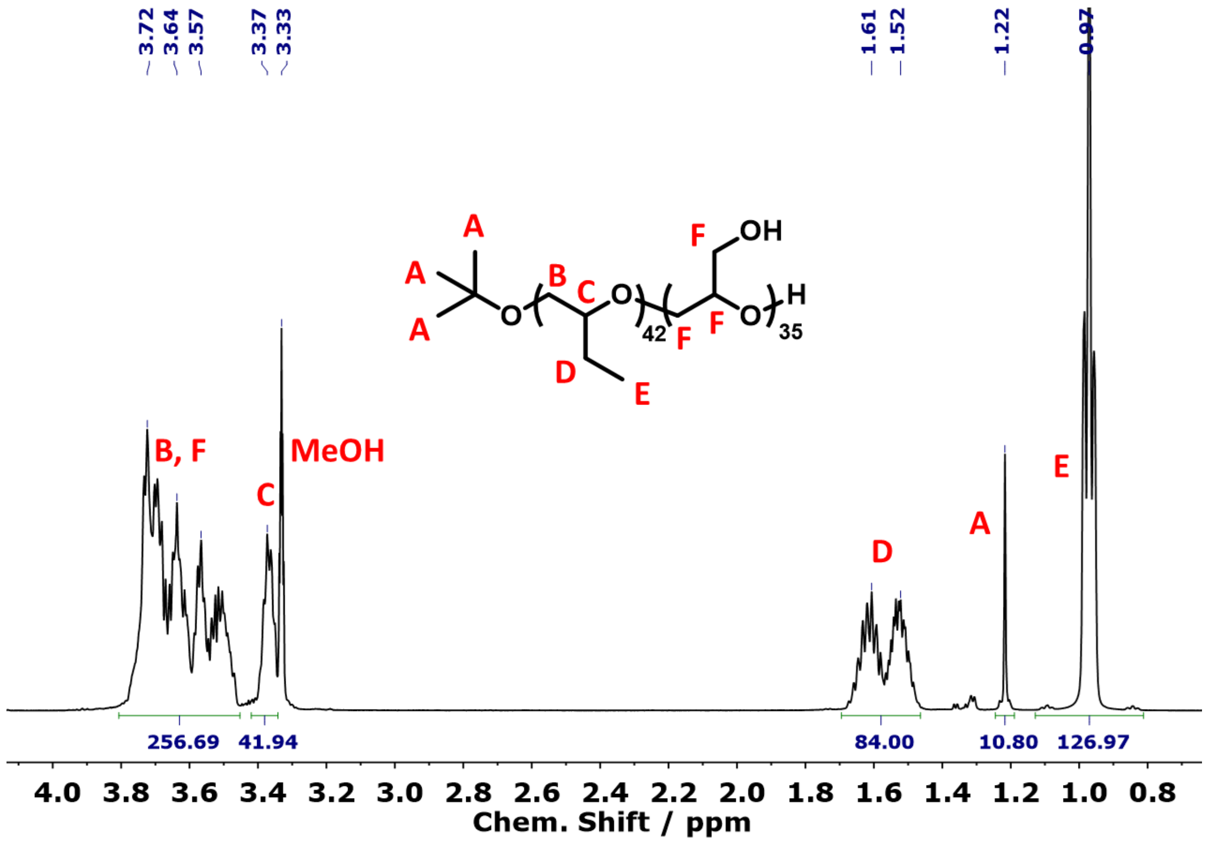

Figure S5: ^{1}H-NMR spectrum of PBO_{42}-*b*-PG_{35} in MeOD.

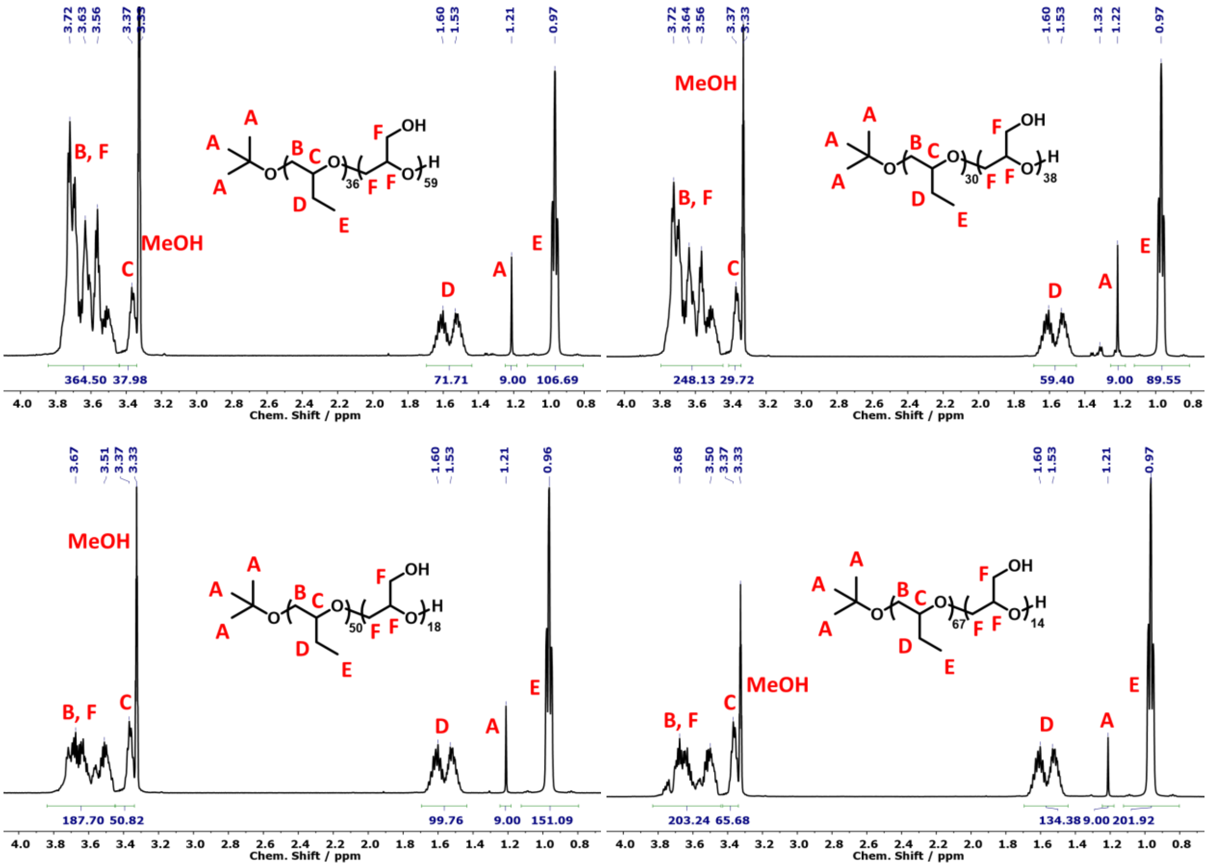

Figure S6: ^{1}H-NMR spectra of PBO_{36}-*b*-PG_{59}, PBO_{30}-*b*-PG_{38} , PBO_{50}-*b*-PG_{18} and PBO_{67}-*b*-PG_{14} in MeOD.

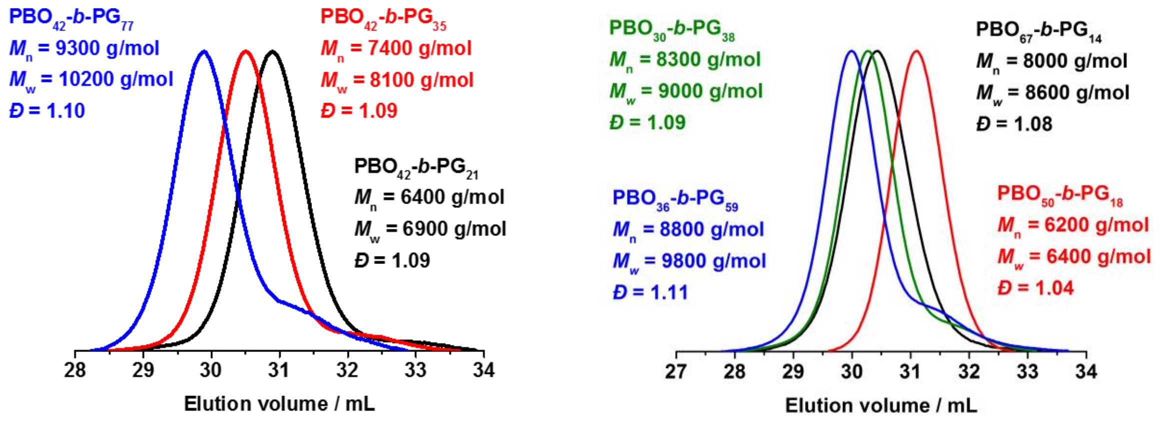

Figure S7: SEC traces of the copolymers forming pure self-assembly phases (left) and of the copolymers forming mixed phases (right) in DMF (RI detector).

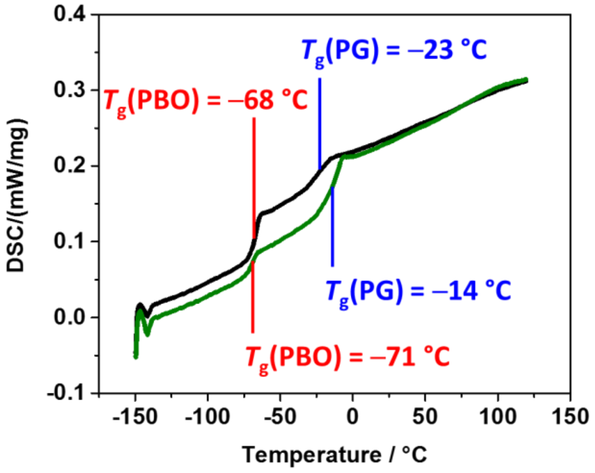

Figure S8: DSC traces of PBO_{42}-*b*-PG_{21} (black) and PBO_{42}-*b*-PG_{77} (green).

2. Self-assembly protocol

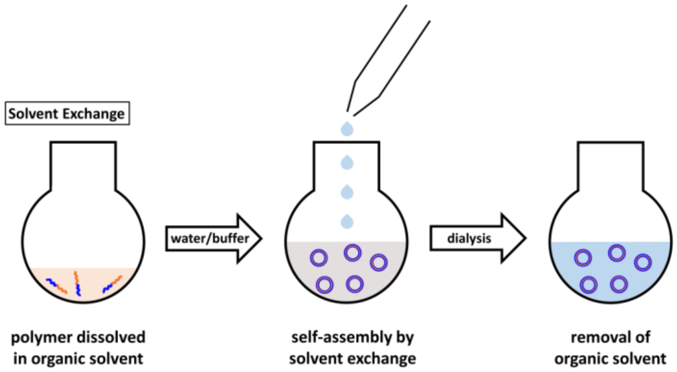

Figure S9: Schematic representation of the self-assembly done *via* solvent exchange.

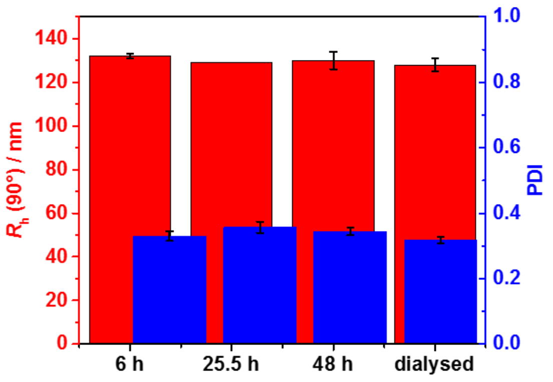

Figure S10: DLS measurements of the self-assembly kinetics into vesicles with PBO_{42}-*b*-PG_{21} showing an apparently finished self-assembly process already after 6 hours after the start of the addition of water.

3. Characterisation of Solvent Switch Self-Assemblies

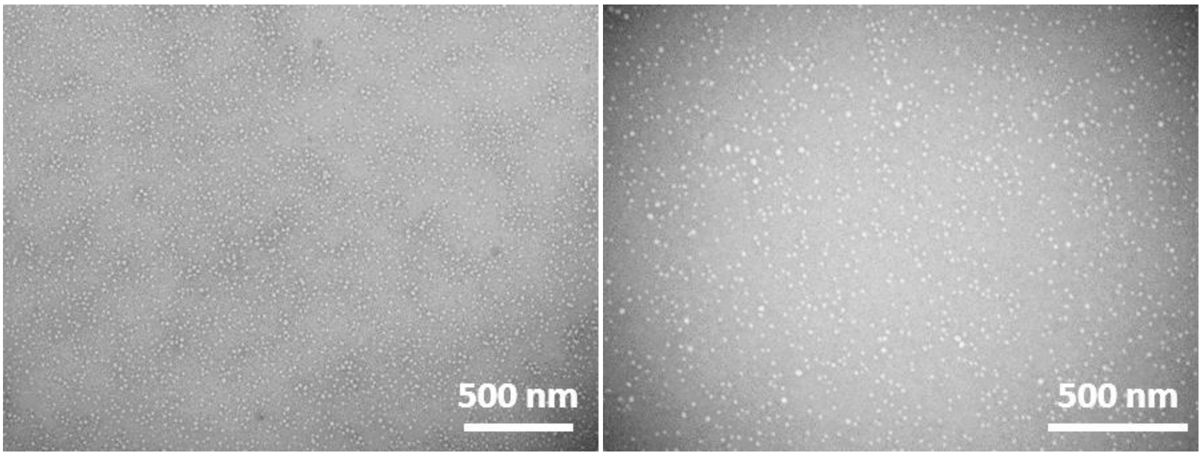

Figure S11: Additional TEM images of micelles formed *via* solvent exchange from PBO_{42}-*b*-PG_{77}.

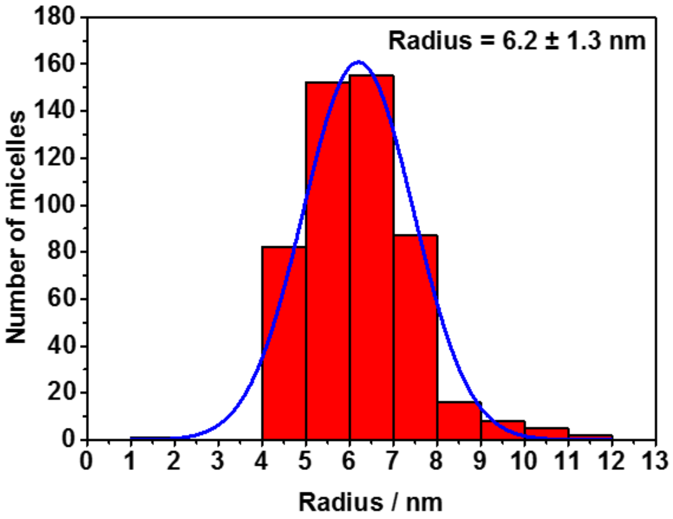

Figure S12: Histogram of the radii distribution of 506 micelles formed *via* solvent exchange from PBO_{42}-*b*-PG_{77}, measured from TEM images by ImageJ.

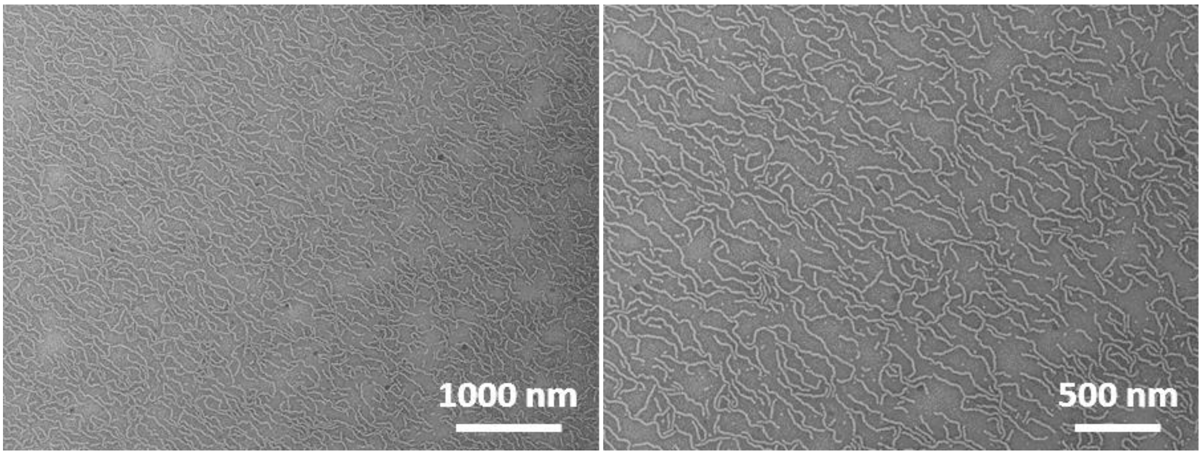

Figure S13: Additional TEM images of worms formed *via* solvent exchange from PBO_{42}-*b*-PG_{35}.

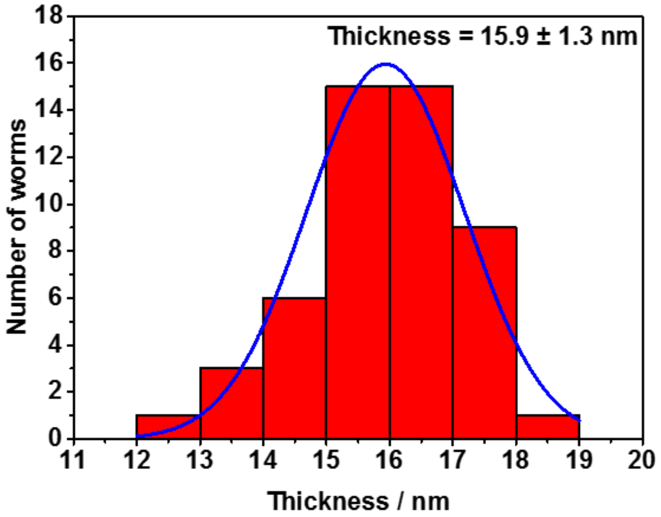

Figure S14: Histogram of the thickness distribution of 50 worms formed *via* solvent exchange from PBO_{42}-*b*-PG_{35}, measured from TEM images by ImageJ.

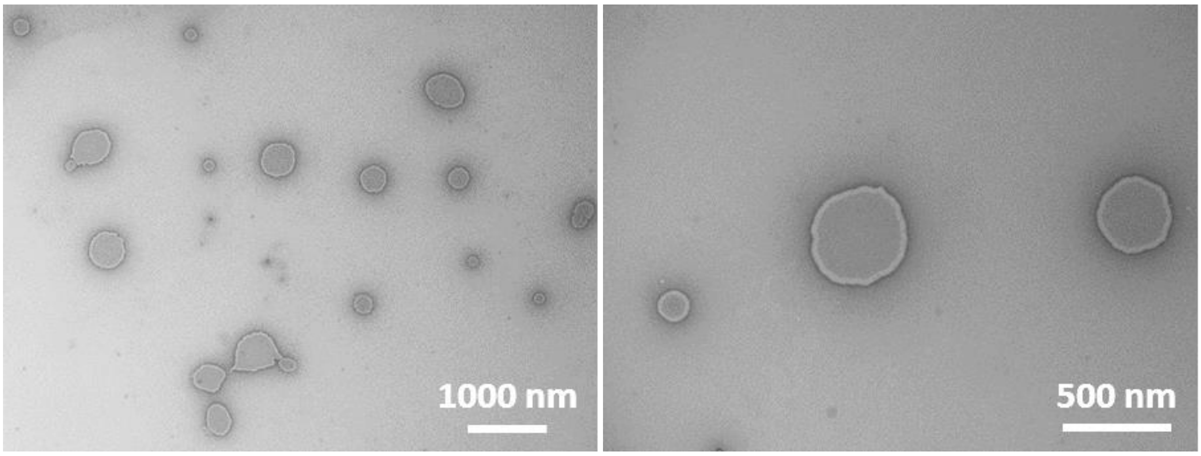

Figure S15: TEM images of polymersomes before extrusion formed *via* solvent exchange from PBO_{42}-*b*-PG_{21}.

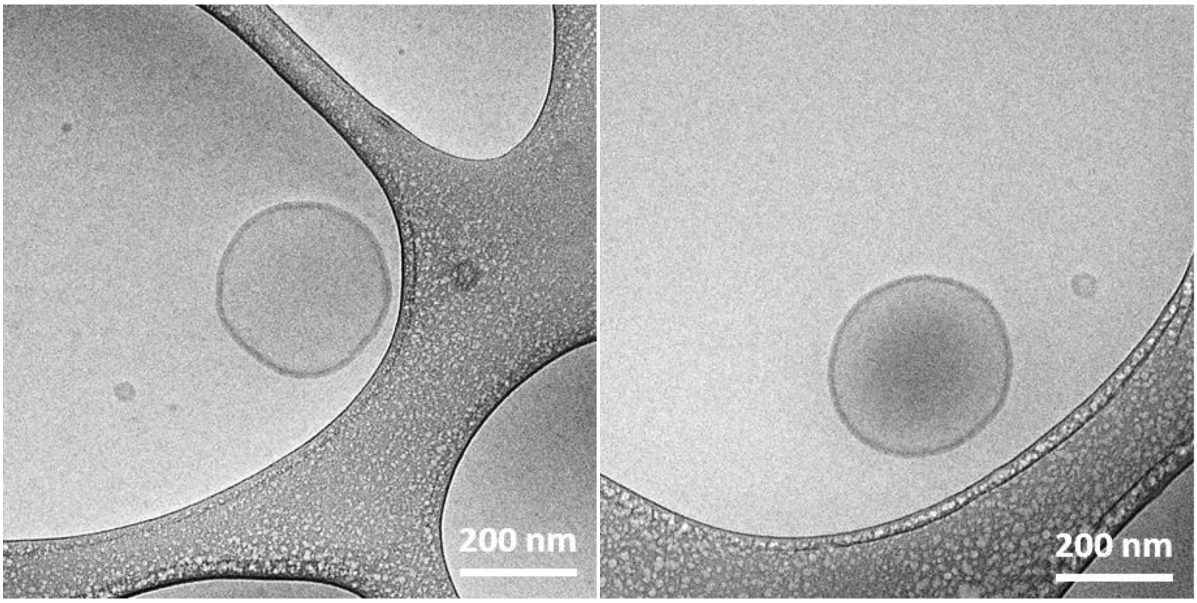

Figure S16: Cryo-TEM images of polymersomes before extrusion formed *via* solvent exchange from PBO_{42}-*b*-PG_{21}.

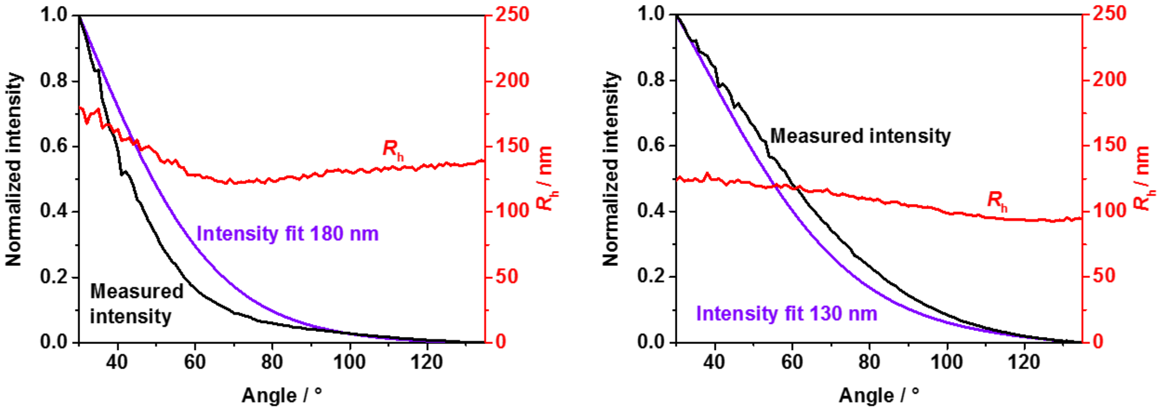

Figure S17: SLS and DLS results for polymersomes formed *via* solvent exchange from PBO_{42}-*b*-PG_{21} before (left) and after (right) extrusion with a 200 nm membrane. The best Mie fit for the intensity curves (black) are shown in violet. When choosing the best fit, we focused on the angular range higher than 90 ° as nanometer-sized structures are more likely to scatter at higher angles. Also the hydrodynamic radius R_h (red) measured by DLS shows a plateau in this range. The radii determined by the intensity fits correlate with the radii of gyration R_g over the equation $R_g^2 = (3/5)R^2$. For the sample before extrusion we calculated R_h = 127 ± 16 nm, R_g = 147 nm and the particle scattering factor with ρ = 1.16. The deviation from the ideal particle scattering factor of 1.0^2 is caused by the size polydispersity of the polymersomes. For the sample after extrusion we calculated R_h = 108 ± 11 nm, R_g = 101 nm and ρ = 0.93. This numbers represent hollow spherical particles, as expected for polymersomes.

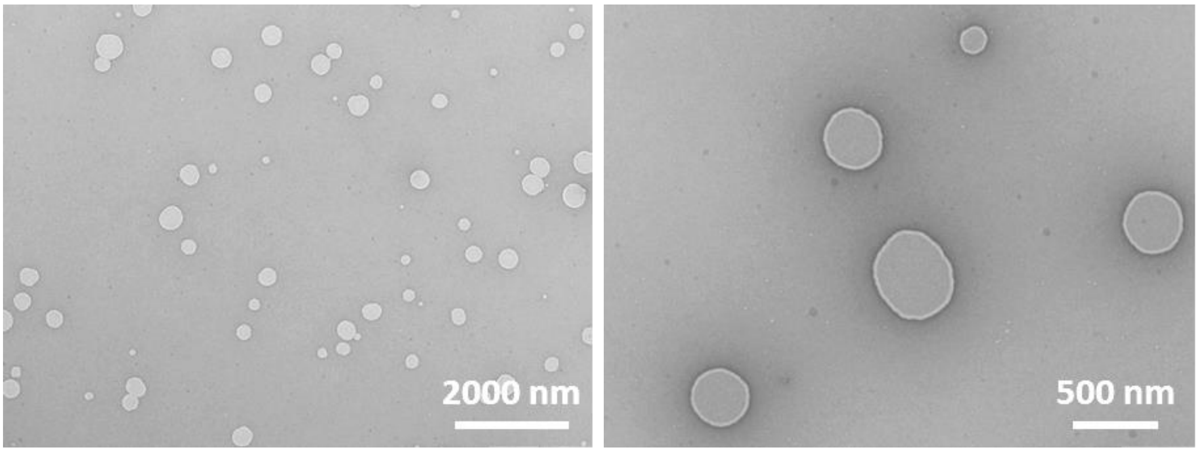

Figure S18: Additional TEM images of polymersomes after extrusion with a 200 nm membrane formed *via* solvent exchange from PBO_{42}-*b*-PG_{21}.

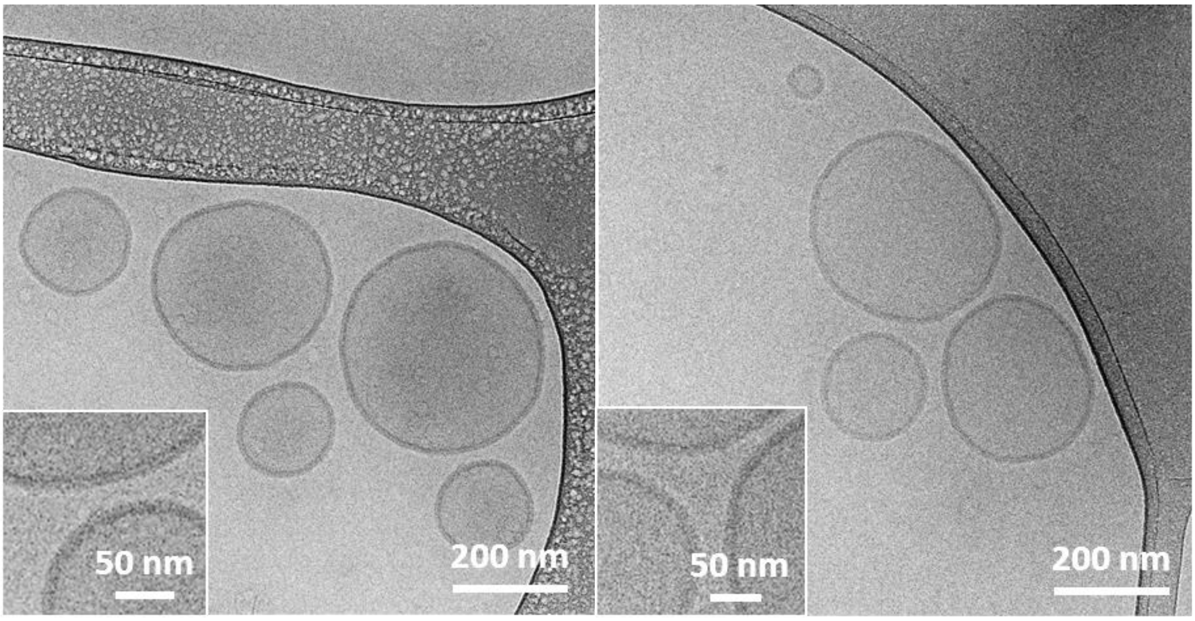

Figure S19: Additional Cryo-TEM images of polymersomes after extrusion with a 200 nm membrane formed *via* solvent exchange from PBO_{42}-*b*-PG_{21}. The insets show the polymer membrane in detail.

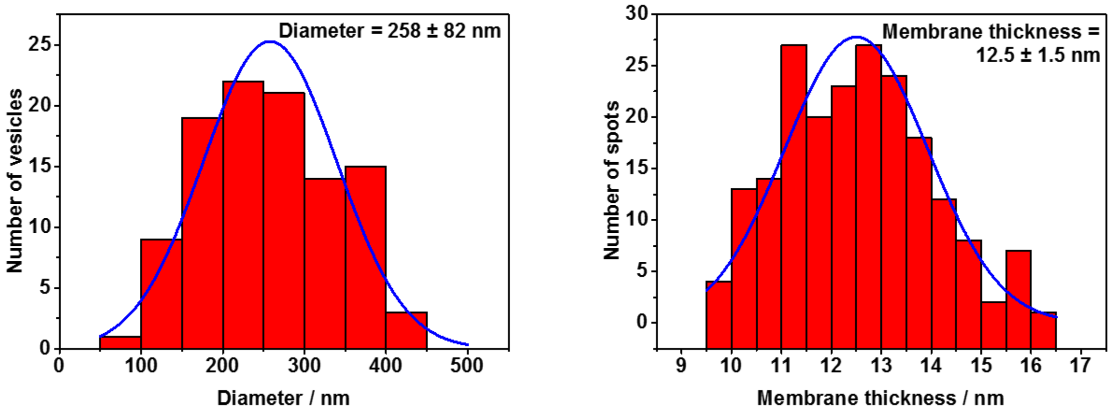

Figure S20: Histogram of the diameter distribution of 104 polymersomes (left) and the membrane thickness of 200 spots on several polymersomes formed *via* solvent exchange from PBO_{42}-*b*-PG_{21} and measured from cryo-TEM images after extrusion with a 200 nm membrane by ImageJ.

4. Effect of Buffer and Different Solvents

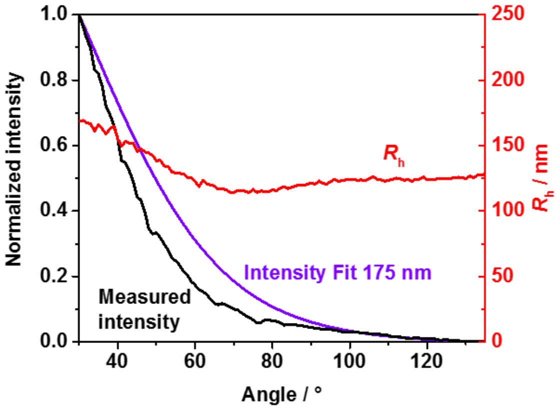

Figure S21: SLS and DLS results of polymersomes formed *via* solvent exchange from PBO_{41}-*b*-PG_{20} in PBS buffer without extrusion. The light scattering data were analysed similar to figure S17, indicating vesicular structures with R_h = 129 ± 15 nm, R_g = 136 nm and ρ = 1.05.

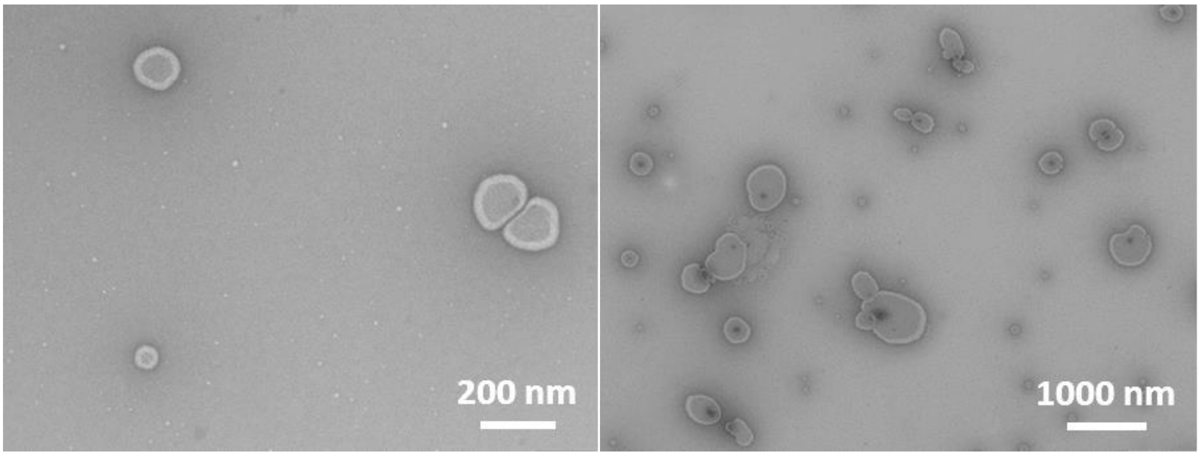

Figure S22: TEM images of polymersomes formed *via* solvent exchange from PBO41-*b*-PG20 in PBS buffer without extrusion.

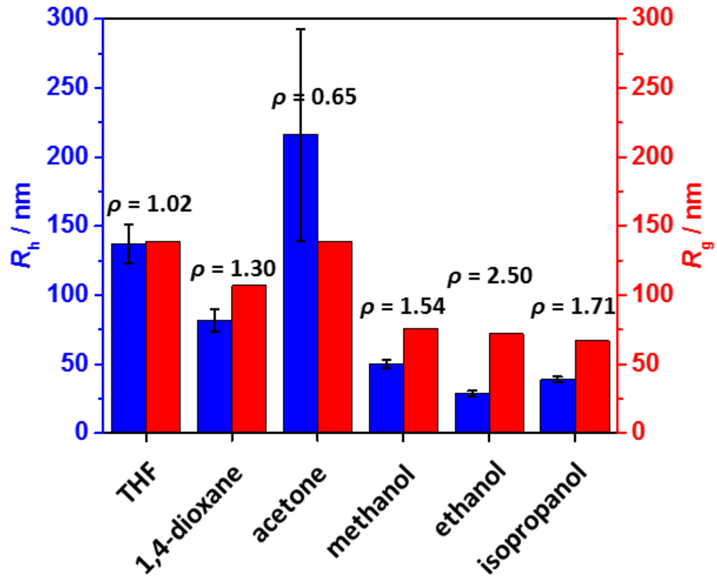

Figure S23: Light scattering results for the self-assemblies formed *via* solvent exchange from PBO42-*b*-PG21 in dependence on different solvents used.

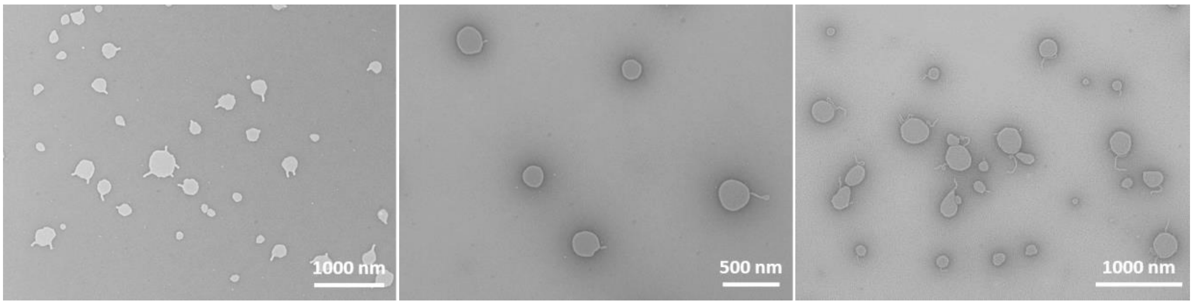

Figure S24: TEM images of the self-assemblies formed *via* solvent exchange from PBO42-*b*-PG21, dissolved in dioxane.

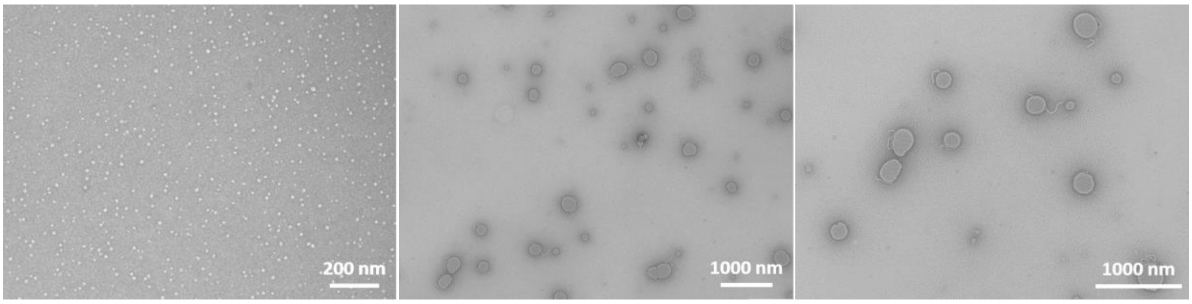

Figure S25: TEM images of the self-assemblies formed *via* solvent exchange from PBO_{42}-*b*-PG_{21}, dissolved in acetone.

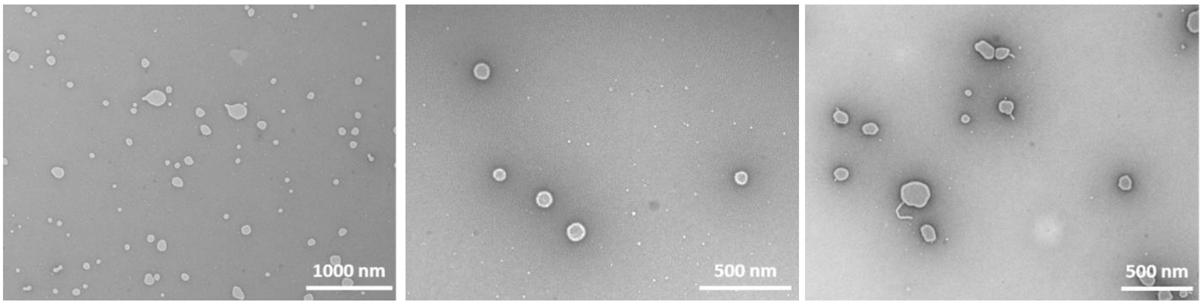

Figure S26: TEM images of the self-assemblies formed *via* solvent exchange from PBO_{42}-*b*-PG_{21}, dissolved in methanol.

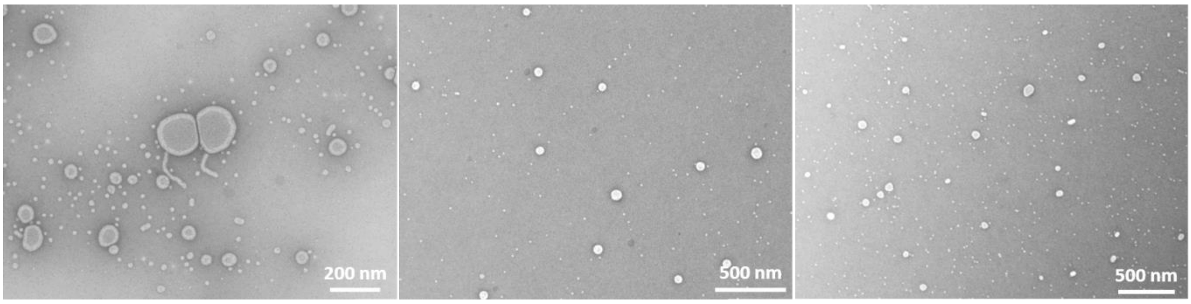

Figure S27: TEM images of the self-assemblies formed *via* solvent exchange from PBO_{42}-*b*-PG_{21}, dissolved in ethanol.

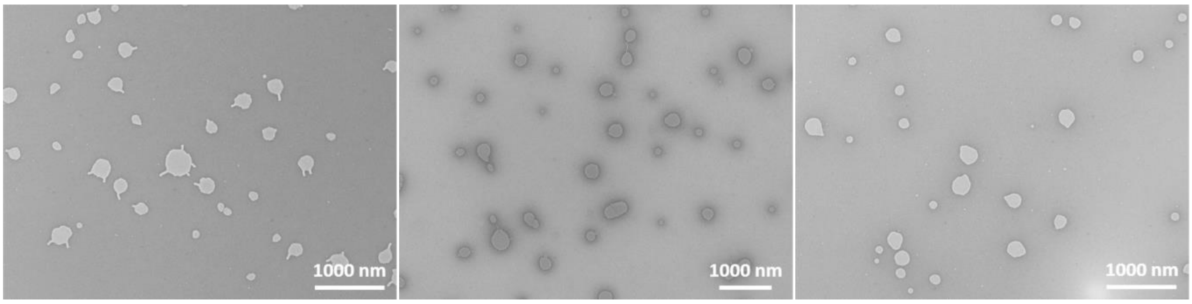

Figure S28: TEM images of the self-assemblies formed *via* solvent exchange from PBO_{42}-*b*-PG_{21}, dissolved in isopropanol.

5. Self-Assembly into GUVs

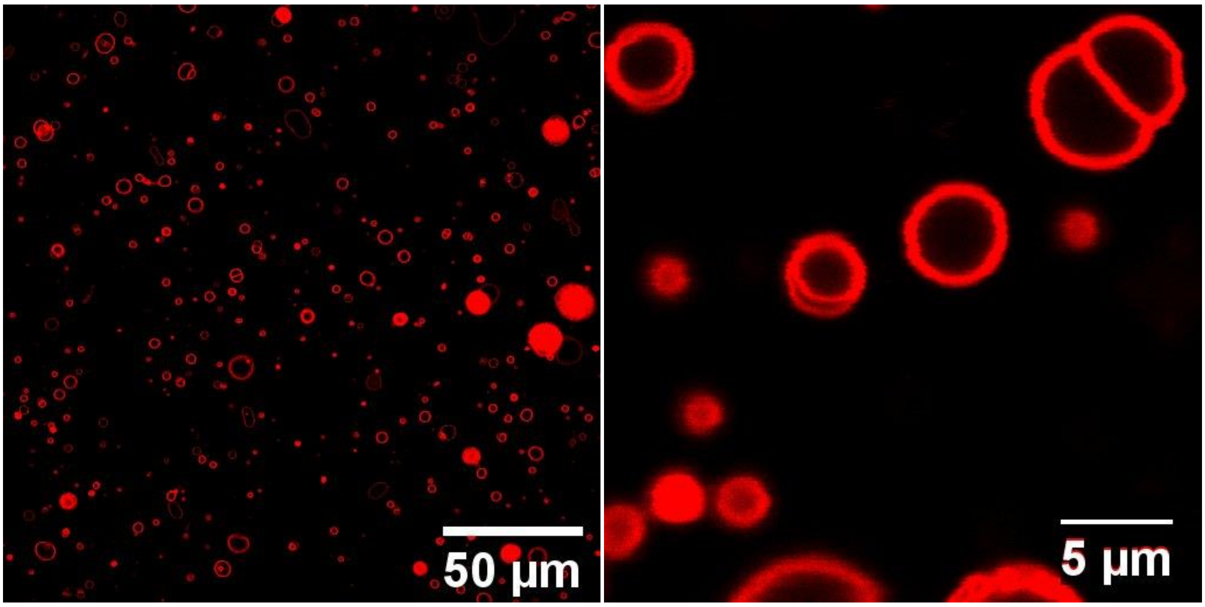

Figure S29: Additional CLSM images of the GUVs formed *via* film rehydration from PBO_{42}-*b*-PG_{21}.

6. Mixed Phases of Intermediate Copolymer Compositions

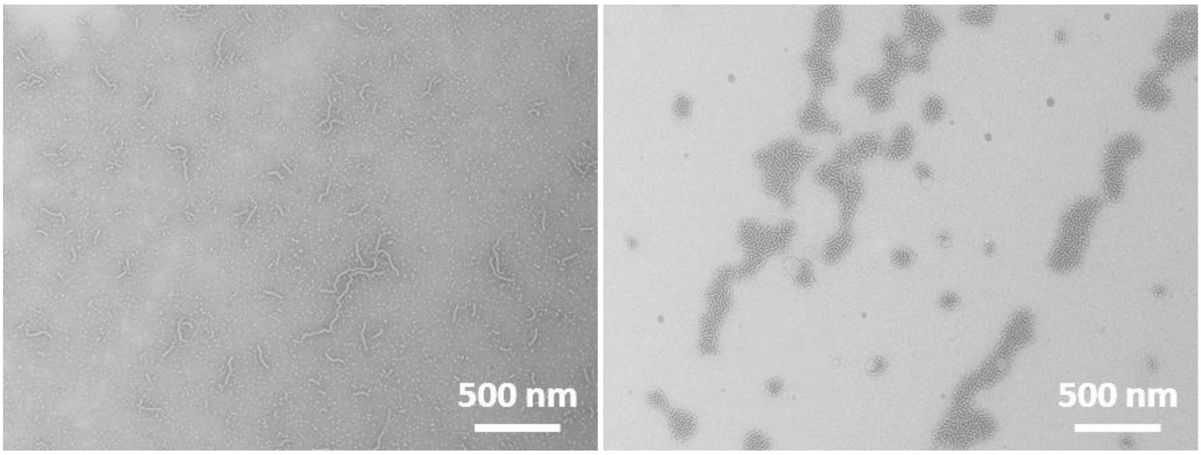

Figure S30: TEM images of the self-assemblies formed *via* solvent exchange from PBO_{36}-*b*-PG_{59} (f = 62%).

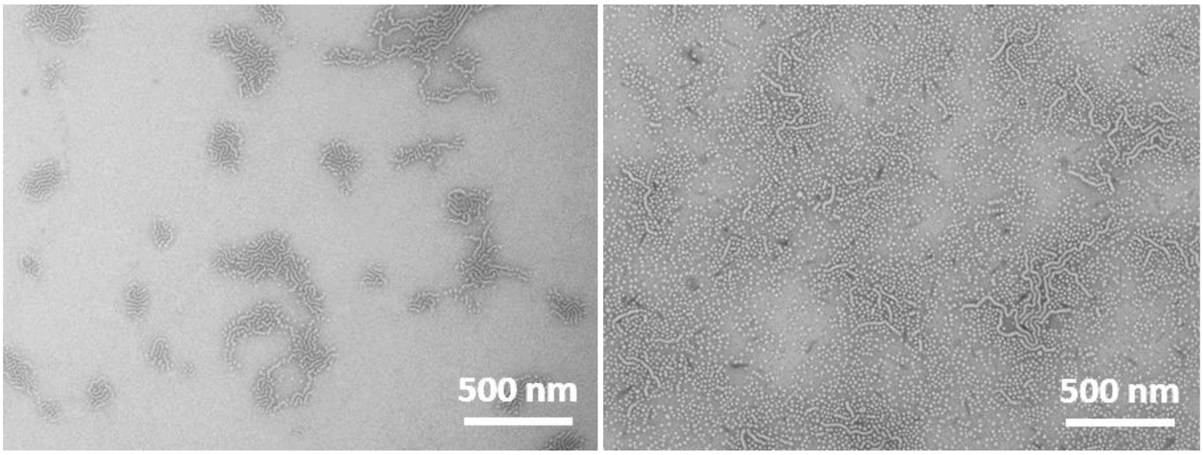

Figure S31: TEM images of the self-assemblies formed *via* solvent exchange from PBO_{30}-*b*-PG_{38} (*f* = 56%).

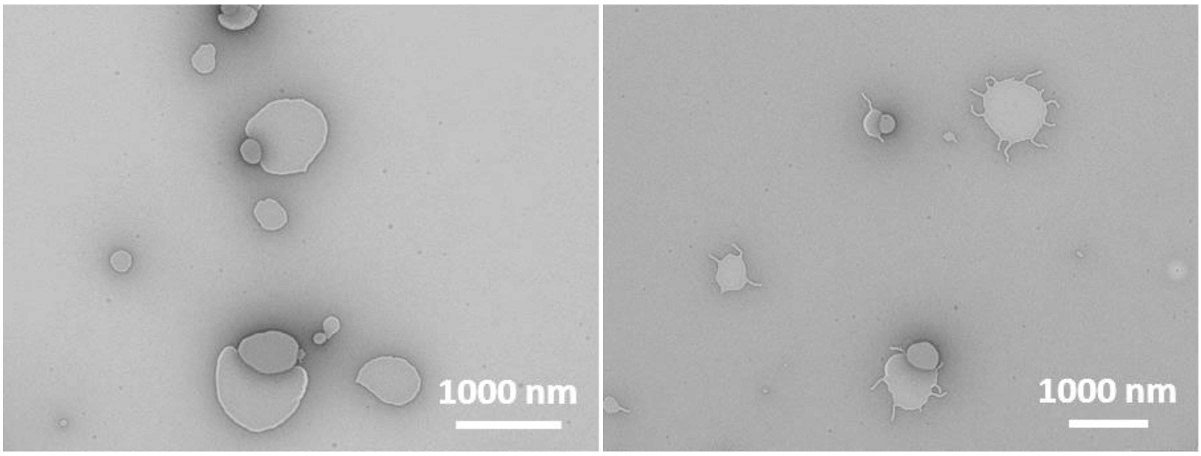

Figure S32: TEM images of the self-assemblies formed *via* solvent exchange from PBO_{50}-*b*-PG_{18} (*f* = 27%).

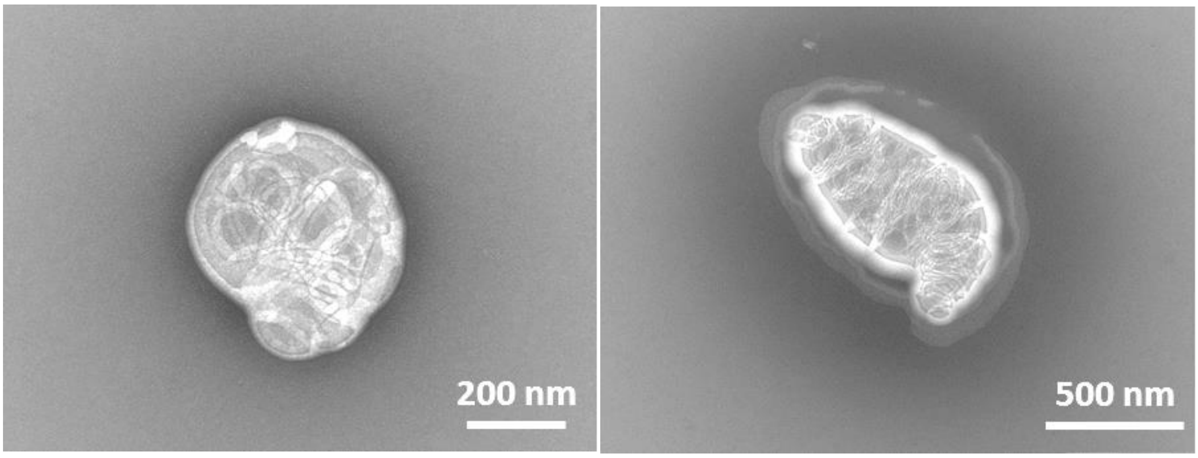

Figure S33: TEM images of the self-assemblies formed *via* solvent exchange from PBO_{67}-*b*-PG_{14} (*f* = 17%).

7. Self-Assembly *via* Film Rehydration

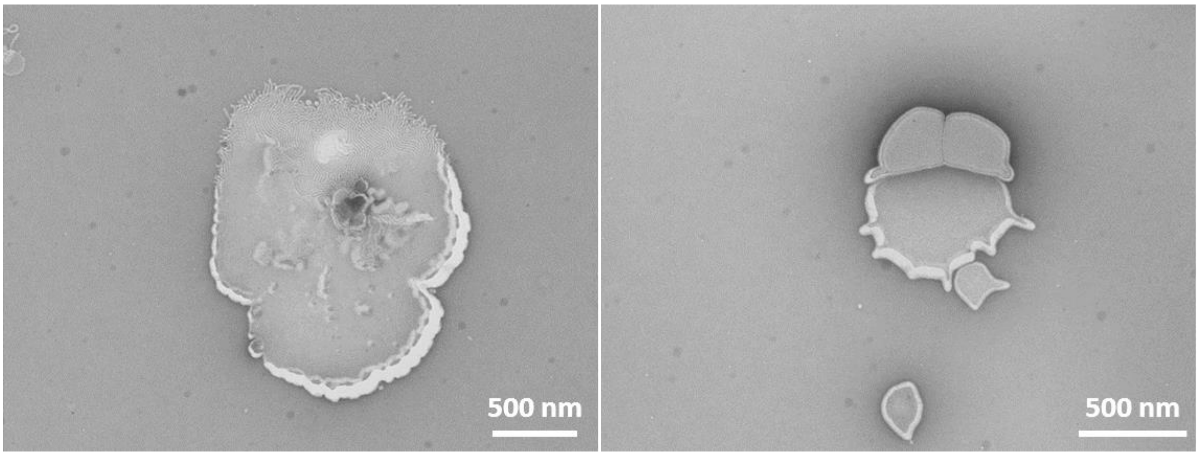

Figure S34: Additional TEM images of the self-assemblies formed *via* film rehydration from PBO_{42}-*b*-PG_{21}.

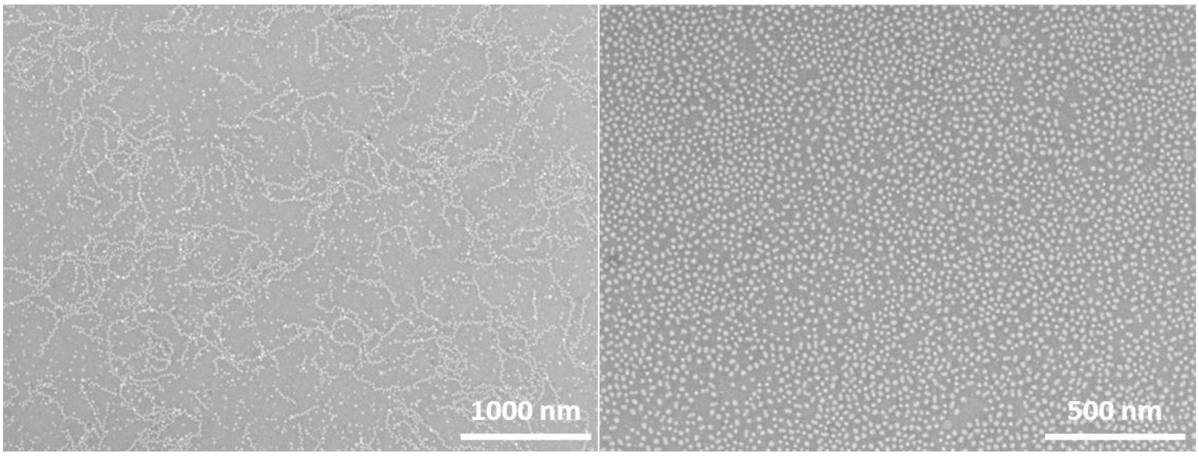

Figure S35: Additional TEM images of the self-assemblies formed *via* film rehydration from PBO_{42}-*b*-PG_{77}.

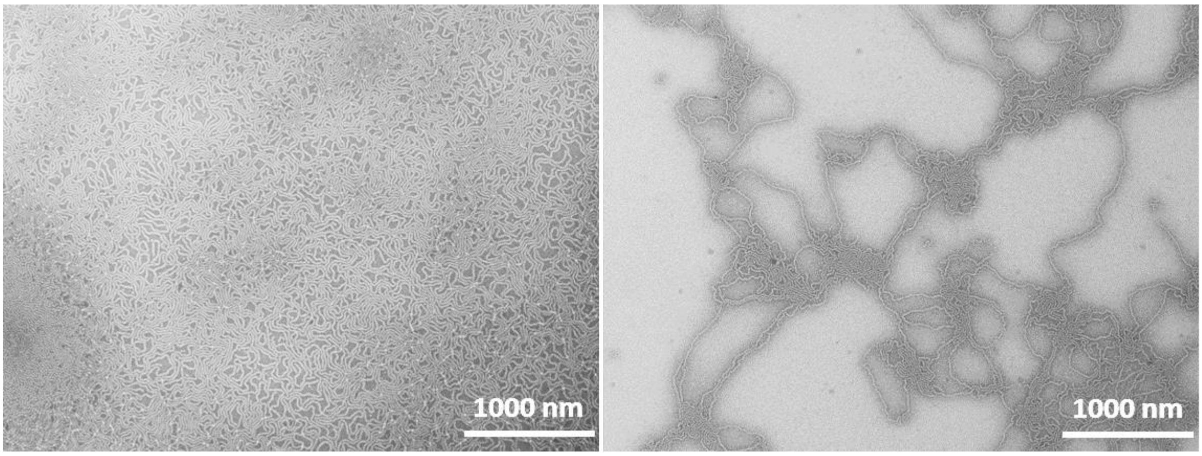

Figure S36: Additional TEM images of the self-assemblies formed *via* film rehydration from PBO_{42}-*b*-PG_{35}.

8. Chain Length in Coil-like and Stretched Conformation

Estimation of the end-to-end distance r in coil-like confirmation of the PBO blocks

r of the PBO blocks in an coiled confirmation was estimated using the average C-C bond length (0.146 nm)[3] and the bond angle of a tetrahedron (109.5 °).The degree of polymerisation was multiplied by 3 as every repeating unit consists of three bonds. The equation is as follows (DP = degree of polymerisation, l = lengths of one bond, θ = bond angle):[4]

$$r^2 = 3 \cdot DP \cdot l^2 \cdot \frac{1 - \cos\theta}{1 + \cos\theta}$$

$$r = \sqrt{3 \cdot 42 \cdot (0.146\, nm)^2 \cdot \frac{1 - \cos 109.5}{1 + \cos 109.5}} = 2.32 \text{ nm}$$

Estimation of the maximum chain lengths l_{max} in stretched confirmation of the PBO blocks

l_{max} between the two ends of the polymer chain for an ideal stretched confirmation of the PBO blocks was estimated using the average C-C bond length (0.146 nm)[3] and the bond angle of a tetrahedron (109.5 °).The degree of polymerisation was multiplied by 3 as every repeating unit consists of three bonds. The equation is as follows (DP = degree of polymerisation, l = lengths of one bond, θ = bond angle):[4]

$$l_{max} = 3 \cdot DP \cdot l \cdot \sin\left(\frac{\theta}{2}\right)$$

$$l_{max} = 3 \cdot 42 \cdot 0.146 \text{ nm} \cdot \sin\left(\frac{109.5}{2}\right) = 15.0 \text{ nm}$$

References

1 A. O. Fitton, J. Hill, D. E. Jane and R. Millar, *Synthesis (Stuttg).*, 1987, **1987**, 1140–1142.

2 W. Burchard, *Static and dynamic light scattering from branched polymers and biopolymers. In: Light Scattering from Polymers. Advances in Polymer Science*, Springer, Berlin, Heidelberg, 48th edn., 1983.

3 J. E. Mark and P. J. Flory, *J. Am. Chem. Soc.*, 1965, **87**, 1415–1423.

4 B. Tieke, *Makromolekulare Chemie*, Wiley-VCH, 3rd edn., 2014.
